# Experiences of infertility-related traumatic events and their association with symptoms of Post-Traumatic Stress Disorder (PTSD) and Complex PTSD: results from a mixed-methods online survey

**DOI:** 10.1093/humrep/deag030

**Published:** 2026-03-12

**Authors:** Sofia Gameiro, Aleksandra Dummer, Lauren Copeland, Gerry McCluskey, Rachel Ross, Derek McLaughlin, Iain McGowan, Hilary Knight

**Affiliations:** School of Psychology, Cardiff University, Cardiff, Wales, UK; Women’s Health Research Wales, Cardiff University, Cardiff, Wales, UK; School of Psychology, Cardiff University, Cardiff, Wales, UK; Women’s Health Research Wales, Cardiff University, Cardiff, Wales, UK; Cardiff School of Sport & Health Sciences, Cardiff Metropolitan University, Cardiff, Wales, UK; British Infertility Counselling Association, York, UK; Fertility Network, East Sussex, UK; School of Nursing & Midwifery, Queen’s University Belfast, Belfast, Northen Ireland, UK; School of Nursing & Midwifery, Queen’s University Belfast, Belfast, Northen Ireland, UK; Fertility Network, East Sussex, UK

**Keywords:** PTSD, complex PTSD, infertility, medically assisted reproduction, trauma-informed care, survey research

## Abstract

**STUDY QUESTION:**

What are experiences of infertility-related traumatic events and are these associated with symptoms of Post-Traumatic Stress Disorder (PTSD) and Complex PTSD (CPTSD)?

**SUMMARY ANSWER:**

Infertility-related trauma results from the interplay between a strong unfulfilled desire for children, negative reproductive events and (lack of) associated care, with 9% and 32% of those reporting a traumatic event meeting criteria for PTSD and CPTSD, respectively.

**WHAT IS KNOWN ALREADY:**

There is worldwide recognition that fertility treatment is highly stressful, but limited understanding of its potential to trigger traumatic responses like PTSD and CPTSD. PTSD is a mental health disorder characterized by reexperiencing the traumatic event, avoidance of traumatic reminders, and a sense of ongoing threat. CPTSD is diagnosed when trauma also leads to difficulty managing emotions, negative self-concept, and disturbances in relationships. We aimed to document infertility-related traumatic events and how these are associated with symptoms of PTSD and CPTSD.

**STUDY DESIGN, SIZE, DURATION:**

Mixed-methods online survey co-produced with and disseminated by the charity Fertility Network UK. Inclusion criteria were being an adult, having suffered from infertility or attended a fertility clinic within the last 5 years, and the ability to read/write English. Eight hundred and sixty-five consented, and 590 (68%, final sample) reported on their most troubling infertility experience.

**PARTICIPANTS/MATERIALS, SETTING, METHODS:**

Most participants were highly educated white heterosexual women in a relationship. The average age was 37.54% had children, 65% were trying to have (more) children, and 30% were undergoing treatment. Infertility-related trauma experiences and symptoms were assessed with the International Trauma Questionnaire (ITQ, Cloitre et al, 2018, 2021), a validated questionnaire that asks people to describe the experience that troubles them the most and to rate how much 18 trauma-related symptoms bothered them in the past month (Likert scale, from 1—none at all to 5—extremely). The ITQ is used to identify participants meeting criteria for PTSD, CPTSD, or both. We replaced ‘experience’ with ‘infertility experience’. Participants also reported on other traumatic experiences, on 12 negative reproductive events (e.g. failed cycle, treatment, [recurrent] miscarriage, stillbirth, and experiences of care).

**MAIN RESULTS AND THE ROLE OF CHANCE:**

Descriptions of infertility troubling experiences yielded 31 categories, 7 themes and 3 meta-themes. Themes showed that suffering and distress are omnipresent in fertility care due to the stressful nature of fertility treatment journeys (e.g. emotional burden, invasive procedures), reproductive loss (e.g. cycle failures, miscarriages), and lack of control (e.g. over access to treatment and its outcomes). This repeated accumulation of distress was compounded by care perceived as dismissive (e.g. insensitive and unsupportive care) and medical trauma (e.g. complications, errors) and had a functional impact at the individual and social level. Fifty-three (9%) participants met criteria for PTSD, 189 (32%) for CPTSD, with 242 (41%) meeting criteria for PTSD or CPTSD. Three-hundred and twenty-eight (56%) participants reported other traumatic events, the 3 most frequent referring to achievement (45%, health and parenthood goals, for example, adoption disruption, recurrent miscarriage), survival (27%, e.g. accidents, traumatic birth) and autonomy (17%, e.g. rape, domestic abuse). The most reported negative reproductive events were unsuccessful cycle (79%), miscarriage (57%), and unsuccessful treatment (52%). Ninety (16%) participants reported that staff discussed trauma with them, 154 (28%) that staff put in place support, and 334 (61%) that the care received made their distress worse. Logistic regression (χ^2^ = 78.600, df = 11, *P* < .001) explained 21.7% of variance in meeting criteria for CPTSD. Participants who met criteria for CPTSD were less likely to have children (OR = 0.461 [0.280, 0.759]), more likely to report a strong child desire (OR = 1.202 [1.036, 1.395]), to have experienced their troublesome infertility experience within the last year (ref category within last year, 1 to 5 years OR = 0.557[0.339, 0.917]), having ended treatment without children (OR = 1.630 [1.051, 2.529], having experienced recurrent miscarriage (OR = 2.2028 [1.109, 3.709]), and reporting that care received made trauma worse (OR = 1.788 [1.124, 2.845]). Background factors (e.g. ethnicity, sexual orientation) were not associated with CPTSD and none of the factors measured were associated with PTSD.

**LIMITATIONS, REASONS FOR CAUTION:**

The survey may have attracted people self-identifying as having experienced traumatic events, but the sample is overall representative of the typical fertility care population and at risk-groups (e.g. ethnicity, sexual orientation, asylum seekers) were unrepresented. Participants meeting criteria for PTSD and CPTSD diagnoses were identified using a self-reporting questionnaire, but which is validated, sound and internationally used.

**WIDER IMPLICATIONS OF THE FINDINGS:**

Many fertility patients have experienced or will experience traumatic events prior to and/or during treatment, and trauma responses can be compounded by poor care and psychosocial contexts. Traumatic events and symptoms must be recognised in clinical practice to prevent (re)traumatising patients, as many will return for treatment and risk re-exposure.

**STUDY FUNDING/COMPETING INTEREST(S):**

None.

**TRIAL REGISTRATION NUMBER:**

Not applicable.

## Introduction

Many patients describe infertility and its treatment—including reproductive losses like miscarriage and recurrent miscarriage—as traumatic. However, few studies have explored if and how fertility care is associated with symptoms of trauma-related clinical diagnoses. The NHS defines traumatic events as events or circumstances perceived as harmful or life-threatening. Most people exposed to traumatic events will experience some level of distress, around 1 in each 3 people will experience lasting negative effects on well-being and functioning (NHS, 2022), and a minority will develop Post-Traumatic Stress Disorder (PTSD) or Complex PTSD (CPTSD). PTSD manifests as re-experiencing, avoidance, and heightened threat perception, while CPTSD includes these symptoms along with disturbances in self-organisation, such as emotion regulation difficulties, negative self-concept, and relational challenges (WHO, 2022). Failure to identify trauma responses in fertility care risks invalidating patients’ lived experiences. For example, emotional dysregulation and increased reactivity are trauma symptoms that can be misinterpreted as ‘difficult’ behaviour, leading to stigma or penalties. It also prevents the provision of mental health care for those more seriously affected (i.e. those with a diagnosis of PTSD or CPTSD). In this mixed-methods online survey study, we investigated infertility-related traumatic experiences, PTSD and CPTSD symptoms, and trauma-related care.

A significant proportion of fertility patients are likely to have experienced one or more traumatic events prior to starting treatment. Around 90% of adults report a history of at least one traumatic experience, including violence, racism, or medical trauma, with 8% subsequently developing PTSD (Lewis *et al.*, 2019). Lifetime PTSD prevalence is 8% in women and 3.4% in men in the USA (Schein *et al.*, 2021). In primary care settings, PTSD prevalence is estimated in a systematic review at 12.5%, with lifetime diagnosis rates ranging from 14.5% to 48.8% (Spottswood *et al.*, 2017). Since fertility treatment primarily targets women, the gendered disparity in trauma exposure is relevant, as women are at greater risk of certain traumatic events (e.g. sexual victimization, intimate partner violence) prior to treatment than are men. For example, in Europe, 26% of women have experienced sexual violence (Li *et al.*, 2023), and sexual assault prevalence ranges from 6.2% to 52.2%, depending on definition and measurement (Dworkin *et al.*, 2021). In some countries, infertility is strongly linked with violence against women. A meta-analysis reported a 36% 12-month prevalence and 47% lifetime prevalence of intimate partner violence among infertile women in low- and middle-income countries (Wang *et al.*, 2022). Given the intrusive nature of fertility procedures, it is essential to consider how treatment may re-trigger trauma. Retriggering happens when a previous trauma response is reactivated, often unintentionally, via exposure to a reminder of the original traumatic event. Indeed, routine exams, personal questioning, invasive touch, and provider authority are seen to trigger memories of past trauma by women undergoing perinatal care (Gordon *et al.*, 2025). However, within fertility care, this is not routinely considered in clinical practice.

Fertility treatment may also induce a trauma response. It can be physically painful, emotionally exhausting, and frightening—all of which are hallmarks of medically traumatizing events (Kazak *et al.*, 2006). For example, although the cause of PTSD was unknown, 6% of women undergoing obstetric/gynaecological procedures in one study met PTSD criteria, and many reported their procedures as traumatizing (Menage, 1993). Reproductive loss—including failed cycles, ectopic pregnancies, and miscarriage—is common and has been associated with subclinical symptoms of post-traumatic stress. One multicentre prospective study found that after miscarriage or ectopic pregnancy, 34% of women reported symptoms of Post Traumatic Stress (PTS, less intense than PTSD, may resolve with time) at 1 month, 26% at 3 months, and 21% at 9 months. 7%, 8%, and 4% of their partners reported symptoms of PTS at the same time intervals (Farren *et al.*, 2021). Additionally, most patients must undergo repeated treatment cycles if they wish to conceive, resulting in prolonged exposure to distress, loss, and uncertainty, which risks leading to cumulative emotional burden (Boivin *et al.*, 2020). This cumulative burden may further worsen even when treatment ends in childbirth, as childbirth can also be traumatising: meta-analysis shows 4% of women meet PTSD criteria postpartum (Ayers *et al.*, 2016).

Some researchers argue that infertility, although not life-threatening, can be experienced as traumatic because it threatens a core life goal: parenthood. This may threaten psychosocial assumptions about life (and fertility) as fair, predictable, and controllable. Ethnographic meta-synthesis (19 qualitative studies, 503 females) shows women see infertility and its treatment as personal reproductive trauma associated with intense stress, grief, insomnia, shame, and guilt, with distress persisting beyond pregnancy (Assaysh-Öberg *et al.*, 2023). Traditional definitions of traumatic events have been critiqued for overlooking cumulative or subjectively distressing experiences such as illness or loss (Boals, 2018; Burstow, 2003). When more inclusive definitions of what counts as a traumatic event are used, infertility can be seen as a threat to psychosocial integrity and basic human needs for affiliation, legacy, and control (especially in pronatalist societies) that could lead to the development of clinically significant trauma responses (Archetti, 2024). Many patients report PTSD and CPTSD-like symptoms during fertility treatment. These include emotional distress triggered by reminders of infertility (PTSD criteria 1, ICD-11), negative self-perception (e.g. shame, worthlessness, CPTSD criteria 2), avoidance (e.g. of social settings involving children, PTSD criteria 2), and emotional dysregulation (e.g. anger, numbness, CPTSD criteria 1) (Gameiro *et al.*, 2015). Subjective experiences of events which are perceived as traumatizing are linked to symptoms of PTSD, regardless of whether they meet diagnostic criteria for PTSD (Maschi *et al.*, 2011).

Whether a fertility patient’s symptoms of PTSD began prior to or during fertility treatment, the emotional consequences (e.g. worsening of symptoms, distress, functional impairment) of the failure to address trauma are similar. Despite this, few studies have investigated trauma responses prior to and during fertility care. Three cross-sectional studies (n = 66, 352, and 173) found that 5%, 37%, and 41% of women undergoing fertility treatment met criteria for PTSD, respectively (Hahn *et al.*, 2021; Roozitalab *et al.*, 2021; Cogendez *et al.*, 2023). The differences may reflect diverse patient profiles, contexts, and assessment tools. PTSD diagnosis in these studies was associated with higher depression, anxiety, and lower quality of life (Yang and Yeo, 2017). One additional study found people with fertility issues had higher PTSD avoidance symptoms compared to those with other reproductive losses, though total PTSD scores were similar. In this study, a provisional diagnosis of PTSD was given to 46% of participants who reported experiencing traumatizing reproductive experiences (Brigance *et al.*, 2023). These studies did not consider CPTSD. Cumulative exposure to traumatic events—including cumulative loss and failed cycles—may lead to the development of CPTSD, yet cumulative fertility-specific risk factors are under-researched.

This survey-based study aimed to investigate infertility-related trauma in patients receiving care in the UK and Ireland. First, we reported on the proportion of participants meeting diagnostic criteria for PTSD, CPTSD, or either—hereafter (C)PTSD, as defined by the World Health Organisation and operationalised in the International Classification of Diseases (WHO, 2022), as this is the classification system used in the UK and Ireland. Second, we documented patients’ perceptions of infertility-related traumatic events and associated care. After we investigated background, fertility, trauma, and care factors associated with meeting criteria for PTSD or CPTSD. Finally, we examined well-being differences between those who met and did not meet PTSD or CPTSD criteria and with or without a clinical diagnosis of PTSD.

## Materials and methods

### Participants

Inclusion criteria were age 18+, experience of infertility or fertility clinic attendance within 5 years, and English literacy. A 5-year window was chosen due to the known lasting impact of traumatic experiences. An a priori power calculation for logistic regression indicated a required sample of N = 445 to detect a medium effect size (0.5) with *α* = 0.05, power = 0.80, assuming a 10% PTSD prevalence—consistent with lifetime prevalence estimates in women—to differentiate individuals meeting versus not meeting PTSD or CPTSD criteria.

### Materials

The Trauma in Infertility Survey (TIS) was a mixed-methods online survey hosted on Qualtrics (Qualtrics, Provo, UT, USA), comprising five sections designed to comprehensively assess participants’ backgrounds, fertility histories, traumatic experiences, fertility care, and well-being.

*The background section* assessed age (in years), gender identity, sex assigned at birth, ethnicity, sexual orientation, education, asylum seeker or refugee status, relationship status, religion, and country of residence.

*The fertility history and treatment section* included a set of questions to assess strength of child desire (*no desire at all* [0] to *a very strong desire* [10]), parental status (childless, children), trying status (trying, never tried, tried and stopped), fertility care status (fourteen options, e.g. never had treatment, waiting to start, undergoing surrogacy with donated eggs or sperm), funding (totally, partially, or self- funded, unsure), past treatment, and total cycles done.

The *about trauma and how it affects you section* began with the International Trauma Questionnaire (Cloitre *et al.*, 2018; Cloitre *et al.*, 2021). The ITQ asks people to think about and describe ‘the *experience that troubles you the most*’ [open-ended text] and specify when it occurred (ranging from <6 months ago to >20 years ago). It then asks people to keep that [infertility-related] experience in mind when rating how much 18 trauma-related symptoms (e.g. “avoiding external reminders of the experience”) had bothered them in the past month (on a 1–5 scale, from none at all to extremely). The ITQ applies a diagnostic algorithm to determine whether respondents meet criteria for PTSD or CPTSD, in line with the International Classification of Diseases (WHO, 2022). Specifically, a diagnosis of PTSD requires the endorsement (i.e. scoring 2 or higher) of one of two symptoms from the symptom clusters of re-experiencing in the here and now, avoidance, and sense of current threat, plus endorsement of at least one indicator of functional impairment. A diagnosis of CPTSD requires, in addition to the PTSD criteria, the endorsement of one of two symptoms from each of the three Disturbances in Self-Organization cluster (affective dysregulation, negative self-concept, disturbances in relationships) and one indicator of functional impairment related to these symptoms. Systematic review provides empirical support for the reliability and validity of the ITQ (Redican *et al.*, 2021). One study using the ITQ in fertility patients reported that 13% of women and 3% of men met criteria for PTSD (Tetecher *et al.*, 2024), though CPTSD was not assessed. In our study, we adapted the ITQ by replacing the term ‘experience’ with ‘infertility experience’. Participants were also asked whether there were other experiences in their life they would consider stressful or traumatic (yes/no), to describe these [open-ended], and report when they occurred. They were then asked if they agreed to be shown a list of 13 reproductive events (e.g. unsuccessful fertility cycle, miscarriage, ectopic pregnancy) and, if so, to indicate which they had experienced and how long ago the most recent occurred.

The *about your reproductive or fertility care section* explored how participants perceived the support received from healthcare professionals (HCPs). Participants reported whether HCPs had discussed infertility-related trauma and ways to cope with it (yes/no), whether they offered support (yes/no/not needed), and whether any part of care worsened their trauma (yes/no/not applicable). Open-ended boxes enabled participants to provide details. Ratings were also collected on comfort levels when discussing trauma or requesting support (from 1—not at all comfortable to 5—extremely comfortable) and on their evaluation of the support received (from 1—extremely bad to 5—extremely good, or not applicable).

The *about your wellbeing section* used the World Health Organisation Wellbeing Index (WHO-5; Topp *et al.*, 2015) to assess recent mental wellbeing. The WHO-5 includes five statements (e.g. ‘I have felt calm and relaxed’) rated on a six-point scale from 0 (none of the time) to 5 (all of the time), covering the previous two weeks. Total scores are scaled from 0 to 100, with higher scores reflecting better well-being. In the UK, the general population mean is 58.6 (56.1 for women, 61.3 for men), and a 10-point difference is considered clinically meaningful. Internal consistency for the WHO-5 in this study was high, with a Cronbach’s alpha of 0.88.

### Procedures

The TIS was disseminated by Fertility Network UK (FNUK) through patient and professional networks, including email lists, closed Facebook and WhatsApp groups, open social media channels, and stakeholder lists comprising health professionals, fertility clinics, government representatives, and allied organisations (e.g. BICA, HFEA, British Fertility Society, Endometriosis UK, Miscarriage Association, Donor Conception Network). The survey ran from October 27 to November 25, 2023. On clicking the link, participants received an information sheet explaining the study’s purpose, voluntary nature, 30-minute duration, and their right to skip questions or withdraw. Participation was open to all, regardless of trauma history. Those who consented accessed the survey. Upon completion, participants were thanked and provided with further study information and a range of support contacts.

### Data analysis

Descriptive statistics were used to characterize the sample across background, fertility and treatment history, and trauma—including proportions meeting criteria for PTSD, CPTSD, or (C)PTSD—and associated care experiences. We considered valid to group patients meeting criteria for PTSD or CPTSD when describing the sample because these share a core set of criteria and risk factors and are perceived by some researchers as falling on a continuum of severity or complexity of trauma response (e.g. Cloitre *et al.*, 2018). Differences between those meeting and not meeting (C)PTSD criteria were tested using chi-square (*χ*^2^) or *t*-tests (*t*), as appropriate. Multivariate logistic regression analyses were conducted to identify background, fertility, trauma, and care (independent) variables associated with PTSD and CPTSD (dependent variables). All variables showing significant univariate associations were entered in each model. Results reported model significance (*χ*^2^), odds ratios (OR) with 95% CIs, and proportion of explained variance (Nagelkerke *R*^2^). ANOVA was used to examine differences in well-being between participants who met and did not meet criteria for PTSD and CPTSD, as well as between those with and without a clinical PTSD diagnosis. These analyses controlled for all variables associated with well-being in univariate tests, specifically education, parental, and trying status. Significance (*F*-test) and effect size (eta squared, *η*^2^) were reported. *η*^2^ values below 0.06 indicate small, between 0.06 and 0.15 moderate, and above 0.15 large effect sizes. A significance threshold of *P* < 0.05 was used.

Open-ended descriptions of ‘the infertility-related experience that troubles you the most’, “support put in place”, ‘support that could be offered’, and ‘aspects of care that made trauma worse’ were analysed separately, using Braun and Clarke (2006) guidelines. These included familiarization, inductive coding, theme and meta-theme generation, and iterative team discussions. The generation of meta-themes was made separately for the question regarding the most troublesome infertility-related experience and experiences of care. Themes and meta-themes were presented with explanatory narratives and illustrative quotes. Coders considered inductive coding was appropriate given that the use of clinical models of trauma would not be appropriate in this non-clinical sample and the absence of clinical diagnoses. For these reasons, no qualitative comparisons were made between participants meeting and not meeting ITQ criteria for (C)PTSD, though this is noted in the illustrative quotes used. Ellipses (…) indicate omitted text; square brackets [] clarify meaning. Open-ended accounts of other traumatic experiences were deductively coded using Kira’s (2001) trauma taxonomy because participants were specifically asked about stressful or traumatic events. Descriptions too vague to classify were labelled ‘Not specified’. Qualitative coding was performed by A.D., S.G., and L.C., with disagreements resolved through discussion.

### Ethical approval

The study was approved by the Cardiff University’s School of Psychology Ethics Committee (EC.21.11.09.6442GA6).

## Results

### Recruitment outcome

A total of 1012 survey accesses were recorded, and 899 participants provided consent. Of these, 34 were excluded due to duplicate responses. From the remaining 865 consenting individuals, 590 reported a traumatic experience or completed the International Trauma Questionnaire (ITQ) and were thus included in the final sample—representing 68% of those who consented.

The final sample of 590 participants had an average survey completion rate of 96.7% (SD = 7.9%, range = 58–100%), with an average completion time of 19 minutes (SD = 9.3, excluding outliers; range = 5–8636 minutes).

Tables 1 and 2 detail participants’ background, fertility, and treatment history. Most were women, white, heterosexual, partnered, and university educated, with an average age of 37. Over half had children, were trying for children, and had undergone an average of four treatment cycles.

**Table 1.** Participants background characteristics (N = 590).

	Total sample N = 590		Did not meet criteria for (C)PTSD n = 334		Met criteria for (C)PTSD n = 242		*t*/χ^2^
**Age**, *M* (SD) [range]**^65 missing^**	37.14 (5.06) [23–65]		37.44 (5.09) [27–65]		36.88 (4.98) [26–53]		1.238
	%	*n*	%	*n*			
**Gender identity**							
Women	98.0	578	98.2	328	97.5	236	
Men	1.5	9	1.5	5	4	1.7	1.461
Prefer to self-describe	0.3	2	0.3	1	0.4	1	
Prefer not to say	0.2	1	0	0	0.4	1	
**Sex assigned at birth^2 missing^**							
Female	98.0	578	98.2	328	97.5	236	1.401
Male	1.6	9	1.5	5	1.7	4	
Prefer not to say	0.2	1	0	0	0.4	1	
**Ethnicity**							
Asian	2.4	14	2.1	7	2.9	7	2.823
Black/African/Caribbean	0.8	5	1.2	4	0.4	1	
Mixed	1.5	9	1.5	5	1.7	4	
Other	0.3	2	0.3	1	0.4	1	
Prefer not to say	0.2	1	0	0	0.4	1	
White	94.7	559	94.9	317	94.2	228	
**Sexual orientation^31 missing^**							
Heterosexual or straight	94.6	529	95.0	304	93.8	212	
Queer (gay or lesbian, bisexual, not sure, prefer to self-describe)	5.0	28	4.4	14	6.2	14	2.288
Prefer not to say	0.4	2	0.6	2	0	0	
**University education^2 missing^**							
No	16.7	99	15.0	50	19.2	46	3.220
Yes	83.0	488	85.0	284	80.4	193	
Prefer not to say	0.2	1	0	0	0.4	1	
**Asylum seeker/refugee^4 missing^**							
No	99.8	585	100	332	99.6	240	1.380
Prefer not to say	0.2	1	0	0	0.4	1	
**Relationship status^3 missing^**							
Single/divorced/separated/widowed	5.8	34	6.0	20	5.4	13	
Partnered/married/cohabiting	93.9	551	93.7	311	94.6	228	0.835
Prefer not to say	0.3	2	0.3	1	0	0	
**Religion^1 missing^**							
No religion	50.8	299	50.8	169	50.8	123	
Christian (all denominations)	45.5	268	45.3	151	45.5	110	
Other	3.1	18	3.0	10	3.3	8	0.521
Prefer not to say	0.7	4	0.9	3	0.4	1	
**Country of residence**							
England	62.0	366	62.6	209	61.6	149	
Northern Ireland	11.7	69	11.7	39	11.6	28	
Republic of Ireland	5.3	31	4.8	16	5.8	14	0.527
Scotland	10.7	63	10.8	36	9.9	24	
Wales	6.4	38	6.3	21	7.0	17	
Other	3.9	23	3.9	13	4.1	10	

Note: 14 people could not be classified as meeting criteria for C(PTSD) or not.

M, mean; (C)PTSD, Post Traumatic Stress Disorder or Complex Post Traumatic Stress Disorder; *t* = *t*-test; χ^2^, Chi-squared test.

* *P* < 0.05,

** *P* < 0.01,

*** *P* < 0.001.

**Table 2.** Participants’ fertility and treatment history characteristics (*N* = 590).

	Total sample N = 590		Did not meet criteria for (C)PTSD n = 334		Met criteria for (C)PTSD n = 242		*t*/χ^2^
**Child desire**, M (SD) [range]^16^**^missing^**	8.75 (2.31) [0–10]		8.35 (2.57) [0–10]		9.31 (1.72) [0–10]		–5.256***
	%	*n*	%	*n*	%	*n*	
**Children^9 missing^**	53.9	313	61.8	204	43.3	103	19.139***
Non-assisted reproduction	13.7	81	14.7	49	12.0	29	0.865
Assisted reproduction of own gametes	34.9	206	41.9	140	26.9	65	13.878***
Assisted reproduction donated gametes	7.8	46	9.6	32	5.0	12	4.29*
Surrogacy	0.3	2	0.3	1	0.4	1	0.053
Adopted	0.3	2	0.3	1	0.4	1	0.053
Fostered	1.2	7	0.9	3	1.2	3	0.159
Stepchildren	0	0	0	0	0	0	–
**Years trying**, *M* (SD) [range]^7 missing^	4.83 (2.67) [0.17–16.33]		4.72 (2.55) [0.17–12]		4.94 (2.79) [0.17–16.33]		–0.784
**Currently trying^13 missing^**							9.403**
No, never tried	1.0	6	1.2	4	0.8	2	
No, tried and stopped	34.3	199	39.6	129	27.4	65	
Yes	64.6	373	59.2	193	71.7	170	
**Years since stopped^22 missing^**	2.81 (3.24) [0–22]		2.91 (3.26) [0–22]		2.66 (3.30) [0.08–20.17]		0.482
**Trying independently^218 missing^**							1.486
No, with partner	98.4	366	99.0	190	97.6	166	
Yes	1.3	5	1.0	2	1.8	3	
Prefer not to say	0.3	1	0	0	0.6	1	
**Fertility care status^38 missing^**							
Never did treatment	1.3	7	1.3	4	1.3	3	
Diagnosis, waiting to start treatment	5.1	28	4.2	13	5.8	13	
Treatment own gametes	20.5	113	15.7	49	27.4	62	37.621***
Treatment donated gametes	4.5	25	4.2	13	5.3	12	
Surrogacy	0.2	1	0	0	0.4	1	
Did treatment, no children	25.0	138	20.1	63	31.0	70	
Did treatment, pregnancy or child	43.5	240	54.6	171	28.8	65	
**Years doing treatment**, *M* (SD) [range]^44 missing^	3.56 (2.64) [0.08–15.5]		3.53 (2.58) [0.08–15.5]		3.56 (2.74) [0.08–15]		–0.126
**Treatment funding^390 missing^**							
Totally publicly funded	22.5	45	22.6	21	20.6	21	
Partially publicly funded	6.5	13	3.2	3	9.8	10	3.695
Self-funded	61.0	122	62.4	58	60.8	62	
Unsure	10.0	20	8.8	11	10.3	9	
**Past fertility treatment**							
OI, IUI, AI with own gametes	38.1	225	38.6	129	37.6	91	0.062
IVF, ICSI with own gametes	74.2	438	74.0	247	75.2	182	0.116
OI, UIU, AI, IVF, ICSI with donated gametes	14.1	83	13.5	45	14.9	36	0.229
Surrogacy with own or donated gametes	1.0	6	1.2	4	0.8	2	0.188
Other (e.g. surgery)	18.5	109	19.2	64	18.6	45	0.029
**Total cycles done,** *M* (SD) [range]^58 missing^	4.12 (3.70) [1–25]		3.90 (3.37) [1–23]		4.32 (3.87) [1–25]		–1.284

Note: 14 people could not be classified as meeting criteria for C(PTSD) or not.

AI, artificial insemination; (C)PTSD, Post Traumatic Stress Disorder or Complex Post Traumatic Stress Disorder; ICSI, intracytoplasmic sperm injection; M, mean; OI, ovulation induction; SD = standard deviation; *t* = *t*-test, χ^2^, Chi-squared test.

* *P* < 0.05,

** *P* < 0.01,

*** *P* < 0.001.

### Proportion of participants meeting criteria for PTSD, CPTSD, or (C)PTSD

Fifty-three (9%) participants met criteria for PTSD, 189 (32%) met criteria for CPTSD, and 242 (41%) met criteria for any of these diagnoses, that is, (C)PTSD. Fourteen participants (2.4%) were not classifiable. A total of 56 (10%) participants reported having received a clinical diagnosis of PTSD. Participants who received a clinical diagnosis of PTSD in the past were more likely to meet criteria for CPTSD (13.5% vs 7.9%, *χ*^2^ = 4.297, *P* = 0.045), but not for PTSD (6.4% vs 10%, *χ*^2^ = 0.648, *P* = 0.606).

### Infertility-related experiences

Three-hundred and twenty-eight participants provided a description of their infertility experience that troubles them the most, corresponding to 55.9% of the total sample. Participants meeting criteria for PTSD or CPTSD were more likely to have had the experience within the last year than participants not meeting criteria (43.1% vs 26.6%, *χ*^2^ = 24.600, *P* < 0.001).

**Figure 1.**
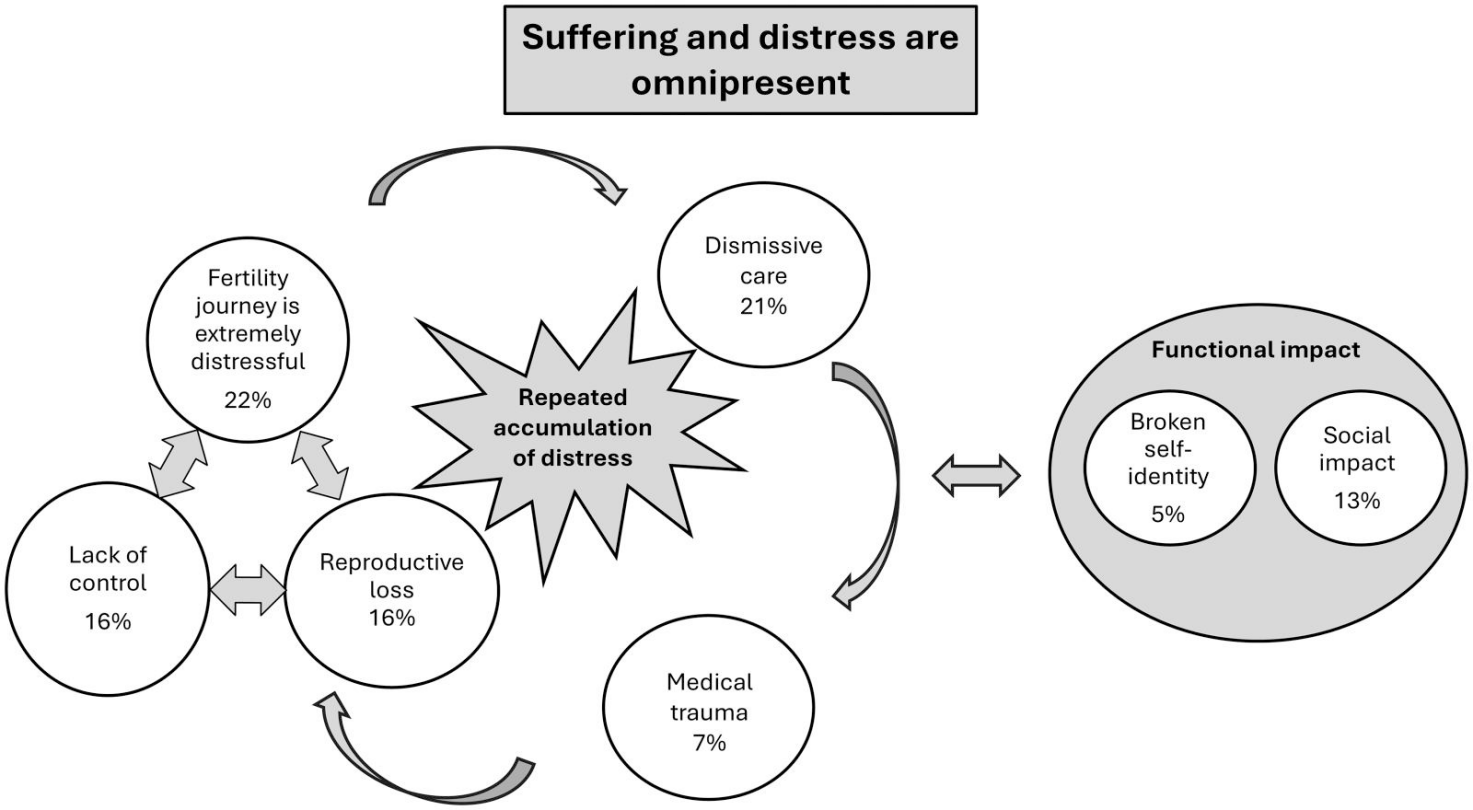
**Thematic map representing the three meta-themes (grey background) and seven themes (white background) identified in qualitative analysis of traumatic experiences reported (N = 590).** For detailed descriptions of each theme and their categories, prevalence and level of endorsement by participants, and illustrative quotes, refer to Supplementary Tables S1, S2, S3, S4, S5, S6 and S7.

Thematic analysis of participants’ narratives yielded 1714 initial codes, which were organized into 31 categories, 7 core themes, and 3 overarching meta-themes. A thematic map illustrating these relationships is shown in Fig. 1 and illustrative quotes are presented in the text. Detailed descriptions of themes, categories, and further illustrative quotes can be found in Supplementary Tables S1, S2, S3, S4, S5, S6, and S7.

The main meta-theme reflects perceptions that *Suffering and distress are omnipresent* in fertility care. This was supported by three interrelated themes: the extremely distressful nature of fertility treatment, reproductive loss, and lack of control. Participants described (in)fertility journeys as extremely distressful due to the ongoing emotional strain, physical burden, and invasive procedures, making treatment feel ‘all-consuming’ (P 560).

‘I felt like I was in a tunnel. I had to force myself to get up each day and function. I returned to work and at the first sign of pressure, I had an anxiety attack and was sent home’. P 141, Met criteria for (C)PTSD

Many spoke of the painful contrast between their hope and effort to have children and the deep disillusionment when treatment failed. Even when IVF resulted in pregnancy, the initial joy often gave way to anxiety. The process left them emotionally exhausted, with many feeling that it had taken over every aspect of their lives.

Fertility journeys were also marked by the (repeated) experience of *reproductive loss*. Participants described loss as beginning with the shock of infertility or diagnosis, followed by repeated cycle failures, miscarriages, stillbirths, and perinatal deaths:

‘Everything about it is traumatizing - physical violation, constant emotional trauma, (…), the absolute utter grief of it. Grief that has nowhere really to go because it’s a grief for something that never was’. P 69, Did not meet criteria for (C)PTSD

‘I still have days where the grief is unbearable. I am unable to talk about my experience without crying and sometimes feel quite isolated. My mum who I have always been quite close to told me I needed to get over it’. P 141, Met criteria for (C)PTSD

Secondary infertility was often overlooked, adding to participants’ pain. Grieving was seen as a central, ongoing part of the journey, yet the depth and duration of suffering were rarely acknowledged by others. Ending treatment without children was experienced as an invisible loss of the future patients had envisioned, compounding feelings of isolation and sorrow.

*Lack of control* was also experienced and stemmed from high treatment costs, unequal NHS funding access, uncertainty despite major sacrifices, prolonged waiting, and the absence of patient-centred care. These structural barriers left participants feeling frustrated and hopeless, compounding the emotional toll of infertility and making the journey feel unpredictable, inequitable, and beyond their influence or control.

‘The uncertainty around whether it would ever happen was crippling. The amount of drugs, time, admin, waiting that you have to do when going through infertility and then undergoing treatment is all consuming and there is still no guarantee’. P 560, Did not meet criteria for (C)PTSD

The second meta-theme reflected the *repeated accumulation of* distress inherent to these three aspects of fertility care, which was compounded by dismissive care and medical trauma. *Dismissive care* reflected perceptions of being treated insensitively or without compassion by HCPs, receiving poor communication, and lacking adequate preparation for treatment (e.g. information about success rates). Participants noted limited emotional support and systemic barriers to empathetic care:

‘The process is extremely cold and harsh, the emotional pressures through the treatment are never addressed properly in the clinics and the recognition of this also in clinic is quite poor’. P 85, Met criteria for (C)PTSD

The increasing marketization of fertility services further eroded trust. These experiences undermined patients’ ability to cope with setbacks and negatively impacted their mental health and overall well-being.

*Medical trauma* stemmed from painful, distressing, and sometimes life-threatening procedures, surgical complications, and care errors that caused delays or inappropriate interventions, potentially reducing the chances of having children. Participants also described discovering new health issues or having long-term conditions exacerbated by treatment. These difficult experiences added complexity to their fertility journey and were seen to be minimised or dismissed by HCPs.

‘With the interruptions in the room, people coming and going and the pain and discomfort I was experiencing I started to cry. The nurse asked why I was crying. At this point I was in a distressed and almost dissociative state. The consultant finally performed the transfer which had taken an hour in total. I left the clinic in tears (…)’. P 80, Met criteria for (C)PTSD

The third meta-theme captured the *functional impact* of infertility at the social and personal level. *Social impact* captured views that the fertility journey is an isolating experience, straining couple communication and intimacy, and causing jealousy or anger toward other people with children. Participants reported limited support, especially from friends and family:

‘I was very fed up, emotionally withdrawn from social situations, I was angry with other people’s birth announcements and most of my friends had babies or kids so I reduced the amount I saw them. I felt life was so unfair’. P 441, Did not meet criteria for (C)PTSD

Social impacts extended to work, where treatment was hard to balance, created additional stress, and often reduced productivity.

The *broken-self* theme captured a sense of shattered identity, marked by self-blame, feelings of inadequacy, shame over unmet social expectations, and a deep detachment from or even hatred toward one’s own body:

‘The news it hadn’t worked again was indescribable. I have never felt that desperate. I screamed for about half an hour and had to be calmed down. These experiences have caused me to truly hate my own body. I’ve never felt so angry, and it is all anger towards myself’. P 54, Met criteria for (C)PTSD

### Other reproductive and life events

Table 3 summarizes participants’ responses regarding other reproductive events. Nearly all (96.4%) agreed to report on reproductive events, with no significant differences between those meeting and not meeting (C)PTSD criteria. The most frequently reported events were one or more unsuccessful fertility cycles, followed by miscarriage and failed fertility treatment. Participants meeting criteria for (C)PTSD were more likely to have experienced unsuccessful fertility treatment, abortion, miscarriage, recurrent miscarriage and less likely to experience health complications after birth than participants who did not meet criteria.

**Table 3.** Other reproductive events reported by participants (N = 590).

	Total sample N = 590		Did not meet criteria for (C)PTSD n = 334		Met criteria for (C)PTSD n = 242		χ^2^
	%	*n*	%	*n*	%	*n*	
**Agreed to report on reproductive events^10 missing^**	96.4	559	96.4	317	96.2	229	0.007
**When reproductive events occurred^22 missing^**							
< 6 months ago	24.6	145	23.3	69	34.1	73	16.362**
6–12 months ago	16.6	98	15.9	47	22.4	48	
1–5 years ago	39.8	235	52.0	154	36.4	78	
5–10 years ago	4.9	29	5.7	17	5.1	11	
10–20 years ago	1.9	11	2.4	7	1.9	4	
>20 years ago	0.3	2	0.7	2	0.0	0	
**Reproductive events**							
Unsuccessful fertility cycles^12 missing^	79.2	441	77.5	245	81.1	185	1.041
Unsuccessful fertility treatment^17 missing^	52.2	288	46.6	146	58.4	132	7.269**
Ectopic pregnancy^26 missing^	9.0	49	9.1	28	8.1	18	0.148
Abortion^28 missing^	9.4	51	6.8	21	13.6	30	6.678*
Miscarriage^22 missing^	56.7	310	51.6	161	62.6	139	6.387*
Recurrent miscarriage^27 missing^	17.7	96	11.8	36	25.1	56	15.996***
Complicated birth^23 missing^	20.7	113	22.3	69	18.8	42	0.922
Stillbirth^20 missing^	2.2	12	1.9	6	2.7	6	0.340
Baby loss^23 missing^	0.9	5	0.3	1	1.4	3	1.844
Health complications during pregnancy^29 missing^	17.0	92	19.3	59	14	31	2.501
Health complications after birth^19 missing^	16.2	89	19.6	61	12.0	27	5.440*
Mental health problems^19 missing^	22.0	121	21.8	68	22.2	50	0.014
Other^45 missing^	13.6	66	11.9	33	16.3	32	1.886
Any of the above^15 missing^	95.8	531	95.6	301	96.5	218	0.276

Note: 14 people could not be classified as meeting criteria for C(PTSD) or not.

* *P* < 0.05,

** *P* < 0.01,

*** *P* < 0.001.

(C)PTSD, Post Traumatic Stress Disorder or Complex Post Traumatic Stress Disorder; χ^2^, Chi-squared test.

Table 4 presents other stressful or traumatic life events, categorized using Kira (2001) taxonomy, along with illustrative quotes. A total of 328 participants (55.9%) reported one or more stressful or traumatic life events, with an average of 1.41 (SD = 0.65). The most prevalent types of events were achievement or self-actualisation—primarily linked to fertility-related losses preventing participants from having (more) children—and survival. Survival traumatic events encompassed baby loss, miscarriage, and life-threatening reproductive and non-reproductive events (e.g. car accidents).

**Table 4.** Other stressful or traumatic life events reported by participants (N = 590).

	Total sample N = 590		Did not meet criteria for (C)PTSD n = 334		Met criteria for (C)PTSD n = 242		χ^2^
	%	*n*	%	*n*	%	*n*	
**Reported other stressful or traumatic life event(s) ^3 missing^**	55.9	328	57.5	191	53.5	129	0.907
Attachment or intimacy	12.4	31	11.3	17	14.0	14	0.393
Autonomy, identity or individuation	17.2	43	18	27	16	16	0.169
Interdependence or disconnectedness	36	90	38.7	58	32	32	1.157
Achievement or self-actualization	44.8	112	41.3	62	50	50	1.822
Survival	27.2	68	18.4	46	22	22	2.276
Not specified	1.6	4	1.3	2	2	2	0.026
Number of traumatic events categories reported, Mean (SE)	1.41 (0.4)		1.41 (0.05)		1.36 (0.06)		0.640
**When other stressful or traumatic life event(s) occurred^12 missing^**	**%**	***n***	**%**	***n***	**%**	***n***	
<6 months ago	14.2	45	12.0	22	18.4	23	4.462
6–12 months ago	21.0	38	10.4	19	14.4	18	
1–5 years ago	28.8	91	29.5	54	26.4	33	
5–10 years ago	17.1	54	19.1	35	14.4	18	
10–20 years ago	18.0	57	19.1	35	17.6	22	
>20 years ago	9.8	31	9.8	18	8.8	11	
**Trauma taxonomy used with illustrative quotes from participants**							
**Category**	**Illustrative quotes**						
Attachment	*Death of loved ones. First relationship breakdown*. P174, Met criteria for (C)PTSD *Divorce resulting in house sale & financial uncertainty*. P356, Did not meet criteria for (C)PTSD *Relationship breakdown with parents Break up with boyfriend Bullying as a child*. P305, Did not meet criteria for (C)PTSD						
Autonomy, identity or individuation	*I have been raped and been in an abusive relationship*. P566, Did not meet criteria for (C)PTSD *Being told by my GP that the only reason I wasn’t conceiving was due to my high BMI*. P550, Did not meet criteria for (C)PTSD *Domestic abuse (emotional) with an ex-partner*. P396, Met criteria for (C)PTSD						
Interdependence or disconnectedness	*Sudden unexpected death of family member*. P355, Did not meet criteria for (C)PTSD *Losing a close friend to suicide*. P485, Met criteria for (C)PTSD *My husband passed away while I was pregnant*. P68, Did not meet criteria for (C)PTSD						
Achievement or self-actualization	*Adoption disruption*. P99, Did not meet criteria for (C)PTSD *Three miscarriages*. P112, Did not meet criteria for (C)PTSD *Recurrent miscarriage*. P584, Did not meet criteria for (C)PTSD						
Survival	*I am in the armed forces and have been through a few difficult experiences*. P45, Met criteria for (C)PTSD *A serious car crash when I was young*. P440, Did not meet criteria for (C)PTSD *Traumatic birth and neonatal hospital stay*. P539, Did not meet criteria for (C)PTSD						
Not specified	*Family life*. P121, Met criteria for (C)PTSD *These were from childhood and I don’t think they affect me now, it did come up a bit when I was struggling with infertility. But I feel fine now*. P388, Did not meet criteria for (C)PTSD *Would rather not say*. P69, Did not meet criteria for (C)PTSD						

Note: 14 people could not be classified as meeting criteria for C(PTSD) or not.

* *P* < 0.05,

** *P* < 0.01,

*** *P* < 0.001.

(C)PTSD, Post Traumatic Stress Disorder or Complex Post Traumatic Stress Disorder; Pn, participant number; χ^2^, Chi-squared test.

### Experiences of reproductive or fertility-related care

**Figure 2.**
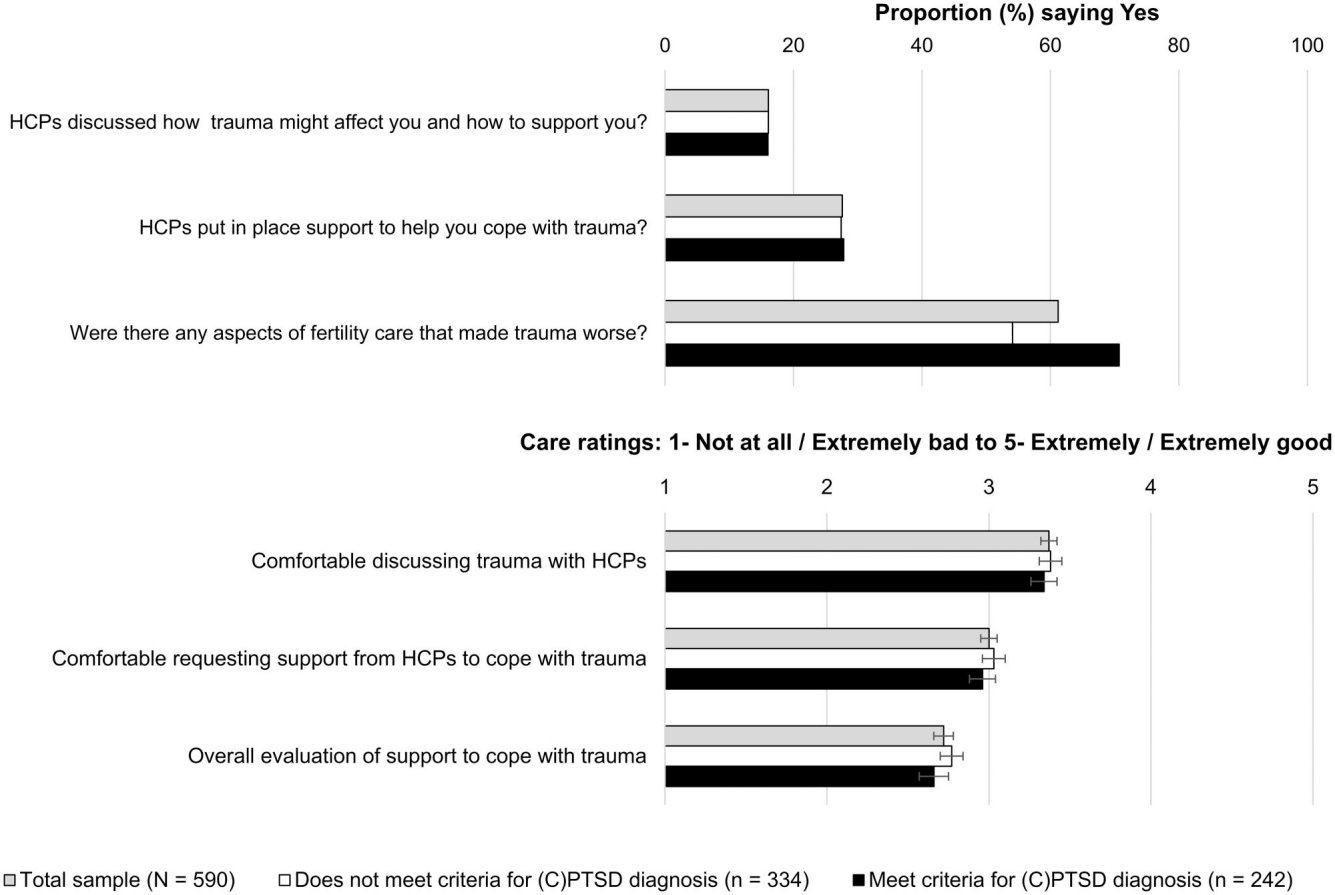
**Experiences of trauma-related care reported by participants (N = 590). Error bars indicate standard error.** (C)PTSD, Post Traumatic Stress Disorder or Complex Post Traumatic Stress Disorder; HCPs, healthcare providers.

Figure 2 presents participants’ ratings of trauma-informed care (TIC) experiences. A minority reported that HCPs discussed trauma (15.9%) or provided support (26.8%), while 17 participants (3.0%) said support was not needed. The majority (61.1%) felt aspects of care worsened their trauma, and 12 (2.2%) said the question was not applicable. Those meeting criteria for (C)PTSD were more likely to report care worsened their trauma than those who did not meet criteria (70.7% vs 54.1%, χ^2^ = 15.742, *P* < 0.001). Likert-type responses indicated participants generally felt uncomfortable discussing trauma or seeking support from HCPs, and their overall evaluation of trauma support was negative, with no significant differences as a factor of meeting criteria for (C)PTSD.

Thematic analysis of narrative responses generated 1326 initial codes, which were grouped into 25 categories, 5 themes, and 4 meta-themes (see Supplementary Fig. S1 for a thematic map). Illustrative quotes are presented in the text, with detailed themes descriptions in Supplementary Tables S8, S9, S10, and S11.

The first meta-theme reflected an unmet need for *psychosocial care*. Participants described limited preparation for the psychosocial impact of infertility and poor access to support, reducing their ability to cope, including when treatment did not work.

‘I have been having fertility treatment for 8 years in total, including with my daughter. No one has ever offered me support with the impact this has had on my mental health or even acknowledged it is hard’. P 183, Met criteria for (C)PTSD

When participants discussed infertility-related distress with HCPs, most reported being offered or referred to counselling and received some form of talking therapy, including CBT, hypnotherapy, mindfulness, or trauma-specific interventions like Eye Movement Desensitization and Reprocessing. Most sessions were short-term, often limited to one, three, or six appointments. HCPs explored topics such as grief, treatment impact, fears for future children, and communication with partners or employers. Participants also discussed non-counselling support, such as social networks or signposting to services (e.g. support groups, bereavement midwives), and that they were rarely offered mental health medication. Participants recommended counselling, support groups, and tailored resources. They suggested clear advertising, accessible referral pathways, and integration of support—especially counselling—into fertility treatment as a standard, rather than optional, part of care.

‘I saw a fertility psychologist before starting treatment and she outlined the steps to take if I need any further support throughout my journey and beyond, the resources available, etc’. P 154, Did not meet criteria for (C)PTSD

The second meta-theme focused on *communication and information provision*. Many participants described care experiences as dismissive, lacking empathy and compassion. Poor language choices, tone, and even microaggressions—such as BMI shaming, ageism, or misogyny—were reported. Patients highlighted a lack of recognition or validation of their emotional and physical needs, which were often minimised or ignored. They also noted limited preparation and inadequate information about treatment outcomes and impacts, leaving them feeling unable to express their needs. Only few participants recalled discussions about expectations for their fertility journey. Participants called for systemic and cultural change in fertility services, including better administrative communication, clearer information, and improved HCP communication styles. From the outset, discussions should address both the physical and mental impacts of IVF, not just success rates. Aligning expectations with reality could help reduce the psychological impact and trauma experienced throughout the treatment process.

‘Unsupportive or unhelpful comments. A constant push to carry on with treatment due to the funding constraints regardless of my wellbeing. Being told by the consultant to put my “big girl pants” on when I said how I was struggling to cope’. P 34, Did not meet criteria for (C)PTSD

‘A clearer understanding of the impact IVF can have whether successful or not and to be told from the beginning the small chance of IVF even working’. P9, Met criteria for (C)PTSD

The third meta-theme captured *organisational aspects of care*. Patients described a lack of resources—funding, time, staff, and training—as well as treatment errors, poor management, and limited privacy, including distressing exposure to pregnant women during reproductive loss, delays and poor-quality care. COVID-19 restrictions further disrupted treatment. Participants recommended that services become more responsive to patient needs, offer clear information and treatment timelines, and ensure easy access to support resources and referral pathways throughout the treatment pathway.

‘Having minimal consultation at the clinic, feeling rushed through the process. I feel that our one shot of funded treatment was wasted due to our lack of understanding of the process and how much it was taken from us. We were deers in the headlights with no one really telling us any information that we could understand or process’. P 351, Met criteria for (C)PTSD

‘Better systems in the first place to prevent a lot of the distress that was caused through long wait times and admin errors’. P29, Met criteria for (C)PTSD

The final meta-theme contrasted the current business-focused model of fertility care with a preferred *patient-centred care* approach. Participants wanted individualized care plans with built-in mental health support and time to address their needs. They also called for IVF records to be accessible across the NHS—from GPs to antenatal services—to ensure continuity of care, recognition of their experiences, and more tailored, informed support throughout their reproductive journey.

‘Because I went to a private clinic my GP was out of the loop- it would be good to have shared care with them’. P175, Did not meet criteria for (C)PTSD

‘Giving me an individualised care plan which I was involved in creating, rather than simply following their usual practice.’. P 32, Did not meet criteria for (C)PTSD

### Factors associated with meeting criteria for PTSD or CPTSD

#### Univariate associations

Differences between participants meeting and not meeting criteria for PTSD or CPTSD—that is, (C)PTSD, were reported in Table 1, Table 2, Table 3, Table 4, and Fig. 2. No significant differences were observed between participants meeting and not meeting criteria for PTSD (data not reported). Compared with participants not meeting criteria for CPTSD, those who met criteria reported a stronger child desire (9.39 vs 8.44, *P* < 0.001), were less likely to have children (36.8% vs 62.4%, *P* < 0.001), more likely to be trying to have children (72.6% vs 60.5%, *P* = 0.017), to indicate their infertility-related traumatic experience and reproductive traumatic events had happened less than a year ago (72.3% vs 54.8%, *P* < 0.001; 60.6% vs 38.9%, *P* < 0.001), and to have experienced failed treatment (61.6% vs 46.7%, *P* < 0.001), abortion (14.5% vs 7.3%, *P* = 0.008), miscarriage (62.4% vs 53.2%, *P* = 0.044), and recurrent miscarriage (26.0% vs 13.2%, *P* < 0.001). They were less likely to have experienced health complications after birth (10.3% vs 19.3%, *P* = 0.008) but more likely to report fertility care made trauma worse (72.7% vs 55.4%, *P* < 0.001).

#### Logistic regressions

No logistic regression was tested for meeting criteria for PTSD given lack of significant univariate associations.

Logistic regression (*χ*^2^ = 78.600, *P* < 0.001) explained 21.7% of variance in meeting criteria for CPTSD. Participants who met criteria for CPTSD were less likely to have children (OR = 0.461 [0.280, 0.759]), more likely to report a strong child desire (OR = 1.202 [1.036, 1.395]), to have experienced their troublesome infertility experience within the last year (ref category within last year, 1 to 5 years OR = 0.557 [0.339, 0.917]), having ended treatment without children (OR = 1.630 [1.051,2.529]), having experienced recurrent miscarriage (OR = 2.2028 [1.109, 3.709]), and reporting that care received made trauma worse (OR = 1.788 [1.124, 2.845]).

### Associations between trauma and well-being

**Figure 3.**
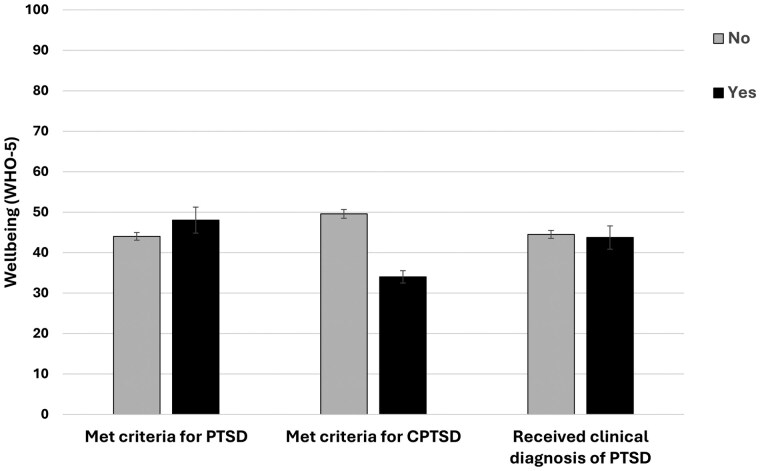
**Psychological well-being of participants according to whether they met criteria for Post Traumatic Stress Disorder (PTSD) and Complex PTSD (CPTSD), and if they received a clinical diagnosis of PTSD.** WHO-5 = World Health Organisation Wellbeing Index. Higher values indicate better well-being. The mean WHO-5 score for females in the UK is 56.1 (Topp *et al*., 2015). Error bars indicate standard error.

Figure 3 displays participants’ psychological well-being scores according to whether they met criteria for PTSD or CPTSD, and whether they received a clinical diagnosis of PTSD. Mean and standard deviation for the whole sample were 44.15 (21.8). One-sample *t*-test with the UK average for females (56.1) indicates moderately lower values (*t* = –12.762, df = 541, *P* < 0.001, Cohen’s *d* = 0.548). Participants who met criteria for CPTSD reported worse well-being scores than those who did not meet criteria (*F* = 68.227, df = 1, *P* < 0.001, *η*^2^ = 0.12). No other statistically significant group differences were observed.

## Discussion

This study provides the most comprehensive account to date of distressful reproductive and infertility events and experiences and their association with trauma symptoms among fertility patients in the UK and Ireland. Our findings indicate that clinically significant trauma symptoms are common in this population. Specifically, 9% of participants met the ITQ diagnostic criteria for PTSD and 32% for CPTSD. The most frequent trauma profile was CPTSD, characterised by difficulty regulating emotions, low self-esteem and self-hatred towards one’s body, partnership challenges and deterioration of ones’ social networks. Distressing experiences were common and seen as omnipresent in the fertility journey for both those who did or did not report symptoms consistent with PTSD or CPTSD. Trauma symptoms were not associated with the ‘amount’ of treatment received. Instead, they were more likely to be reported among patients with a very strong desire for children who were (repeatedly) confronted with reproductive loss or medical trauma within the last year, instead of their desired child, and whose distress was not met with sufficiently empathetic care. The severity of distress experienced may be reflected in the clinically significant lower psychological well-being reported by participants meeting criteria for CPTSD. Findings highlight the need to better understand trauma (symptoms, clinical diagnoses) in the context of fertility care via implementing further and more rigorous research to inform how HCPS can recognize, evaluate, and support patients experiencing these.

The proportion of participants meeting criteria for PTSD and CPTSD in this study (9% and 32%) is considerably higher than UK adult population estimates (5.3% and 12.9%) using the same measure (Cloitre *et al.*, 2018). The proportion of participants meeting criteria for PTSD is lower than observed in other health-related populations: 17.4% in people with cancer (Hansen *et al.*, 2021) and 20.4% in individuals with chronic pain (Siqveland *et al.*, 2017). The proportion of participants meeting criteria for CPTSD is also lower than in trauma and mental-health service samples collected in the UK (41.9% and 53.1%, Karatzias *et al.*, 2016; Hyland *et al.*, 2017) but higher than non-UK samples using the same measure (9.3% and 17.3%, Kazlauskas *et al.*, 2018; Lueger-Schuster *et al.*, 2018). The dominant trauma profile was CPTSD, reflecting complex emotional, cognitive, and relational disruptions consistently reported in infertility research (Verhaak *et al.*, 2007; Greil *et al.*, 2010; Gameiro *et al.*, 2015) that are typically described as stress- or grief-related in much of the infertility literature. Although our study is unable to determine the cause of CPTSD symptoms and clinical diagnoses can only be assigned via performing a clinical interview, results highlight that some symptoms of distress reported by fertility patients are trauma related. Qualitative and quantitative data show that CPTSD symptoms in fertility patients are not linked to the amount of treatment but arise when individuals with a strong desire for children face repeated reproductive loss without sufficient support or empathetic care. Taken together, this evidence likely reflect participant self-selection in survey research, paired with the distinct psychosocial impact of infertility, which has been described by patients as being as stressful as cancer (Domar *et al.*, 1993) and is known to have a unique severe, prolonged, cumulative, but often not recognized emotional burden. These findings support patients’ perceptions of infertility and its treatment as personal reproductive trauma (Assaysh-Öberg *et al.*, 2023). They also support conceptualisation of infertility as a (non-life-threatening) traumatic event, in which potential for trauma is closely linked to the threat it poses to parenthood and assumptions about fertility as predictable and controllable (Olson, 1994). At this point, we must acknowledge the past, largely unsupported, claims that Borderline Personality Disorder (BPD) is associated with infertility, and the ongoing debate between the overlap of CPTSD and BPD (Cloitre *et al.*, 2014; Owczarek *et al.*, 2023). While the latter needs further investigation, this study’s participants did not report many BPD symptoms, such as disturbed patterns of thinking (e.g. hallucinations), impulsive behaviour (e.g. reckless and irresponsible activities), and unstable relationships (e.g. frantic efforts to prevent being left alone). What is relevant for fertility care is that participants are reporting symptoms of CPTSD and requesting appropriate support to manage these.

Distressing reproductive events were highly prevalent in this sample. Consistent with IVF literature, over half of participants reported unsuccessful cycles (79%) and treatment (52%). Miscarriage (57%) and recurrent miscarriage (18%) were far more common in this study sample than in other studies reporting on miscarriage in women of similar age (15% and 2–5%, respectively) (Larsen *et al.*, 2013, Sunkara *et al.*, 2014). Notably, a third of participants (33.9%) reported negative reproductive events within the last year, and this was linked to CPTSD symptoms. Combined with the finding that CPTSD diagnosis is linked to a strong desire for children, this suggests many patients may pursue further treatment while meeting diagnostic criteria for CPTSD. Less often considered is that some patients enter fertility care already carrying symptoms consistent with PTSD or CPTSD. In our sample, 56% reported prior traumatic life events, with nearly 30% experiencing threat to life and 35% reporting that event happened within the past year. Undergoing fertility treatment in the context of unresolved distress from prior trauma exposure likely contributes to the chronicity and complexity of trauma symptoms. Care models must account for how treatment may interact with prior trauma exposure (be it infertility or non-infertility related). This is especially relevant as the UK and Ireland fertility patient population becomes more diverse. Single women and same-sex couples seeking treatment tripled and doubled, respectively, from 2011 to 2022, and Asian patients now account for 15% of IVF cycles (HFEA, 2023), and minoritized groups are more likely to face adversity and trauma exposure (Craig *et al.*, 2020; Maguire-Jack *et al.*, 2020). Unfortunately, like much of the field, our sample mostly reflects the experiences of white heterosexual patients. Finally, fertility staff may vicariously experience patients’ trauma. One study with 1095 obstetricians and gynaecologists working in the UK found two-thirds reported exposure to a traumatic work-related event and, of these, 18% reported clinically significant PTSD symptoms that were associated with lower job satisfaction, emotional exhaustion and uptake of sick leave. This is an area for future research (Slade *et al.*, 2020).

Overall, findings suggest that traditional frameworks of stress and coping may be inaccurate or incomplete to understand and address patient distress. If future studies confirm that PTSD and/or CPTSD are common among fertility patients, this will significantly impact how care is understood and suggest that such care be delivered in a trauma-informed manner. While stress and grief often follow an adaptive, though non-linear, recovery process with appropriate support (Djelantik *et al.*, 2022), exposure to traumatic events can lead to chronic emotional dysregulation, altered self-perception, persistent avoidance, and even increased mortality risk (Nilaweera *et al.*, 2023). Given that only around 56% of UK patients who undergo treatment achieve a live birth and that the postpartum period is a high-risk time for mental health issues (Meltzer-Brody *et al.*, 2017) the potential for co-morbid or worsened mental health outcomes is considerable. The psychological impact of unresolved trauma symptoms is considerable. In this study, participants meeting CPTSD criteria had WHO-5 scores more than 10 points lower than those not meeting criteria—a clinically significant difference (Topp *et al.*, 2015). Qualitative accounts confirmed the severity and wide-reaching effects of trauma, describing profound emotional suffering, exhaustion, hopelessness, and a loss of meaning in life, with negative impacts across personal, relational, and work domains. This highlights the need to recognise and address trauma symptoms and experiences in fertility care. From the patient perspective, more empathic, sensitive and responsive care with an integrated approach to mental health during patients’ fertility pathway, reflected at the organisational (e.g. information provision, articulation with other NHS services) and individual level (i.e. staff recognize the traumatic potential of infertility and its treatment, their wide-ranging impacts on patients, and provide person-centred support) can make a significant difference. Clinics should explore adopting TIC, an approach that recognises trauma’s effects, identifies its signs, and creates a culture of safety while avoiding re-traumatisation (Substance Abuseand Mental Health Services Administration, 2014). TIC in fertility care needs to reflect the specific context of treatment (Lawson and Swanson, 2024) and should be co-produced with staff, patients, and stakeholders. While primarily patient-focused, TIC can also support staff.

This mixed-methods study offers a detailed view of infertility-related distress and the frequency of PTSD and CPTSD in an infertility mostly female patient population. The sample was large enough for robust analysis, but recruited mostly online through our charity partner, likely attracting people identifying as having experienced infertility as particularly distressing. Clinically significant symptoms of PTSD and CPTSD were assessed using the self-report, validated ITQ. A recent study found that adding clinical checks to clarify participants’ understanding of ITQ items reduced prevalence estimates by 1.6% for PTSD and 4.6% for CPTSD (a relative drop of 29.6% and 48.4%, respectively) (Shevlin *et al.*, 2025). Well-being was assessed at the end of the survey, and responses were likely primed by the previous reporting on traumatic events. This suggests our PTSD and CPTSD estimates and distress severity reports may be inflated. However, the sample broadly reflects the typical research-active fertility care population and men and important at-risk groups—such as ethnic minorities, LGBTQ+ individuals, and asylum seekers—were underrepresented. Future research should be more inclusive and disaggregate results for these different groups.

The key implication of this study is the urgent need for more rigorous research into the presence of PTSD and/or CPTSD in diverse infertility patient populations. Existing survey-based studies show wide variability in PTSD and CPTSD estimates, likely inflated by self-selection, while underrepresenting higher-risk groups (e.g. minoritised patients, those with high risk for prior trauma exposure). To accurately establish prevalence, prospective studies with consecutive clinic recruitment, repeated assessments, and diagnostic interviews to assess the cause and symptoms of PTSD and/or CPTSD are essential, particularly to capture CPTSD. Secondly, as in other areas of gynaecology, fertility staff must recognise the traumatic potential of infertility and its treatment, and the wide-ranging impacts these have on patients, and clinics can adopt TIC.

In conclusion, distressing and potentially traumatizing experiences are common in fertility care. Staff must acknowledge that any patient may have a history of trauma. Not all patients will want to disclose this, and they do not have to. Staff do not need to know details about patient’s history, they just need to ask patients what reasonable adjustments they need in their care and endeavour to always provide sensitive care. These simple measures provide a good foundation to prevent (re)traumatising patients, particularly as many will return for treatment and risk re-exposure. The field needs to discuss how specific TIC practices should look like in fertility care.

## Supplementary Material

deag030_Supplementary_Figure_S1

deag030_Supplementary_Table_S1

deag030_Supplementary_Table_S2

deag030_Supplementary_Table_S3

deag030_Supplementary_Table_S4

deag030_Supplementary_Table_S5

deag030_Supplementary_Table_S6

deag030_Supplementary_Table_S7

deag030_Supplementary_Table_S8

deag030_Supplementary_Table_S9

deag030_Supplementary_Table_S10

deag030_Supplementary_Table_S11

## Data Availability

Given the sensitivity of the topic, the team decided the data should not be openly shared, but it can be shared on a reasonable request to the corresponding author.

## References

[deag030-B2] Archetti C. Infertility as trauma: understanding the lived experience of involuntary childlessness. Cult Med Psychiatry 2024;48:940–960. 39003684 10.1007/s11013-024-09871-7 PMC11570559

[deag030-B3] Assaysh-Öberg S , Borneskog C, Ternström E. Women’s experience of infertility & treatment—A silent grief and failed care and support. Sex Reprod Healthc 2023;37:100879. 37356208 10.1016/j.srhc.2023.100879

[deag030-B4] Ayers S , Bond R, Bertullies S, Wijma K. The aetiology of post-traumatic stress following childbirth: a meta-analysis and theoretical framework. Psychol Med 2016;46:1121–1134. 26878223 10.1017/S0033291715002706

[deag030-B5] Boals A. Trauma in the eye of the beholder: objective and subjective definitions of trauma. J Psychotherapy Integr 2018;28:77–89.

[deag030-B6] Boivin J , Harrison C, Mathur R, Burns G, Pericleous-Smith A, Gameiro S. Patient experiences of fertility clinic closure during the COVID-19 pandemic: appraisals, coping and emotions. Hum Reprod 2020;35:2556–2566. 32761248 10.1093/humrep/deaa218 PMC7454659

[deag030-B7] Braun V , Clarke V. Using thematic analysis in psychology. Qual Res Psychol 2006;3:77–101.

[deag030-B8] Brigance CA , Kim S-R, Kashubeck-West S. Mean comparisons of trauma symptoms between a reproductive trauma sample and a normative sample: toward a trauma-informed practice. Psychol Trauma 2023;15:1164–1171. 36972102 10.1037/tra0001468

[deag030-B9] Burstow B. Toward a radical understanding of trauma and trauma work. Violence Against Women 2003;9:1293–1317.

[deag030-B10] Cloitre M , Garvert DW, Weiss B, Carlson EB, Bryant RA. Distinguishing PTSD, complex PTSD, and borderline personality disorder: a latent class analysis. Eur J Psychotraumatol 2014;5:25097. 10.3402/ejpt.v5.25097 PMC4165723 25279111

[deag030-B11] Cloitre M , Hyland P, Prins A, Shevlin M. The international trauma questionnaire (ITQ) measures reliable and clinically significant treatment-related change in PTSD and complex PTSD. Eur J Psychotraumatol 2021;12:1930961. 34211640 10.1080/20008198.2021.1930961 PMC8221157

[deag030-B12] Cloitre M , Shevlin M, Brewin CR, Bisson JI, Roberts NP, Maercker A, Karatzias T, Hyland P. The International Trauma Questionnaire: development of a self‐report measure of ICD‐11 PTSD and complex PTSD. Acta Psychiatr Scand 2018;138:536–546. 30178492 10.1111/acps.12956

[deag030-B13] Cogendez E , Kumru P, Soysal S, Ozkaya E, Devranoglu B, Tozkir E, Sanverdi I. Evaluation of psychological distress in infertile women who underwent ART cycle during the COVID-19 pandemic. Gynaecol Obstet Reprod Med 2023;29:54–62.

[deag030-B14] Craig SL , Austin A, Levenson J, Leung VWY, Eaton AD, D’Souza SA. Frequencies and patterns of adverse childhood events in LGBTQ+ youth. Child Abuse Negl 2020;107:104623. 32682145 10.1016/j.chiabu.2020.104623

[deag030-B15] Djelantik AMJ , Robinaugh DJ, Boelen PA. The course of symptoms in the first 27 months following bereavement: a latent trajectory analysis of prolonged grief, posttraumatic stress, and depression. Psychiatry Res 2022;311:114472. 35248806 10.1016/j.psychres.2022.114472 PMC9159380

[deag030-B16] Domar AD , Zuttermeister PC, Friedman R. The psychological impact of infertility: a comparison with patients with other medical conditions. J Psychosom Obstet Gynaecol 1993;14:45–52. 8142988

[deag030-B17] Dworkin ER , Krahé B, Zinzow H. The global prevalence of sexual assault: a systematic review of international research since 2010. Psychol Violence 2021;11:497–508. 34737898 10.1037/vio0000374 PMC8562086

[deag030-B18] Farren J , Jalmbrant M, Falconieri N, Mitchell-Jones N, Bobdiwala S, Al-Memar M, Tapp S, Van Calster B, Wynants L, Timmerman D et al Differences in post‐traumatic stress, anxiety and depression following miscarriage or ectopic pregnancy between women and their partners: multicenter prospective cohort study. Ultrasound Obstet Gynecol 2021;57:141–148. 33032364 10.1002/uog.23147

[deag030-B19] Gameiro S , Boivin J, Dancet E, de Klerk C, Emery M, Lewis-Jones C, Thorn P, Van den Broeck U, Venetis C, Verhaak CM et al ESHRE guideline: routine psychosocial care in infertility and medically assisted reproduction-a guide for fertility staff. Hum Reprod 2015;30:2476–2485. 26345684 10.1093/humrep/dev177

[deag030-B20] Gordon J , Hunter A, Callanan F, Kiely C, Grealish A. An integrative review exploring womens’ experiences of Retraumatization within perinatal services. J Midwife Womens Health 2025;70:32–49. 10.1111/jmwh.13662 PMC11803493 39036988

[deag030-B21] Greil AL , Slauson-Blevins K, McQuillan J. The experience of infertiilty: a review of recent literature. Sociol Health Illn 2010;32:140–162. 20003036 10.1111/j.1467-9566.2009.01213.x PMC3383794

[deag030-B22] Hahn C , Caraway J, Hansen K, Ranum E. Exposure to traumatic events, emotion regulation, and traumatic stress among infertility patients: a moderation analysis. Human Fertil 2021;24:136–143. 10.1080/14647273.2019.1593517 PMC6774900 30938554

[deag030-B23] Hansen M , Vægter HB, Cloitre M, Andersen TE. Validation of the Danish International Trauma Questionnaire for posttraumatic stress disorder in chronic pain patients using clinician-rated diagnostic interviews. Eur J Psychotraumatol 2021;12:1880747. 34025921 10.1080/20008198.2021.1880747 PMC8128127

[deag030-B24] HFEA. Ethnic diversity in fertility treatment 2021. Preliminary UK ethnicity statistics for IVF and DI treatment, storage, and donation. 2023.

[deag030-B25] Hyland P , Shevlin M, Brewin CR, Cloitre M, Downes A, Jumbe S, Karatzias T, Bisson JI, Roberts NP. Validation of post‐traumatic stress disorder (PTSD) and complex PTSD using the International Trauma Questionnaire. Acta Psychiatr Scand 2017;136:313–322. 28696531 10.1111/acps.12771

[deag030-B26] Karatzias T , Shevlin M, Fyvie C, Hyland P, Efthymiadou E, Wilson D, Roberts N, Bisson JI, Brewin CR, Cloitre M. An initial psychometric assessment of an ICD-11 based measure of PTSD and complex PTSD (ICD-TQ): evidence of construct validity. J Anxiety Disord 2016;44:73–79. 27776256 10.1016/j.janxdis.2016.10.009

[deag030-B27] Kazak AE , Kassam-Adams N, Schneider S, Zelikovsky N, Alderfer MA, Rourke M. An integrative model of pediatric medical traumatic stress. J Pediatr Psychol 2006;31:343–355. 16093522 10.1093/jpepsy/jsj054

[deag030-B28] Kazlauskas E , Gegieckaite G, Hyland P, Zelviene P, Cloitre M. The structure of ICD-11 PTSD and complex PTSD in Lithuanian mental health services. Eur J Psychotraumatol 2018;9:1414559. 33680347 10.1080/20008198.2017.1414559 PMC7874935

[deag030-B29] Kira IA. Taxonomy of trauma and trauma assessment. Traumatology (Tallahass Fla) 2001;7:73–86.

[deag030-B30] Larsen EC , Christiansen OB, Kolte AM, Macklon N. New insights into mechanisms behind miscarriage. BMC Med 2013;11:154–110. 23803387 10.1186/1741-7015-11-154 PMC3699442

[deag030-B31] Lawson A , Swanson A. Trauma-Informed Reproductive Healthcare. Springer, 2024.

[deag030-B32] Lewis SJ , Arseneault L, Caspi A, Fisher HL, Matthews T, Moffitt TE, Odgers CL, Stahl D, Teng JY, Danese A. The epidemiology of trauma and post-traumatic stress disorder in a representative cohort of young people in England and Wales. Lancet Psychiatry 2019;6:247–256. 30798897 10.1016/S2215-0366(19)30031-8 PMC6384243

[deag030-B33] Li L , Shen X, Zeng G, Huang H, Chen Z, Yang J, Wang X, Jiang M, Yang S, Zhang Q et al Sexual violence against women remains problematic and highly prevalent around the world. BMC Womens Health 2023;23:196. 37101173 10.1186/s12905-023-02338-8 PMC10134525

[deag030-B34] Lueger-Schuster B , Knefel M, Glück TM, Jagsch R, Kantor V, Weindl D. Child abuse and neglect in institutional settings, cumulative lifetime traumatization, and psychopathological long-term correlates in adult survivors: the Vienna Institutional Abuse Study. Child Abuse Negl 2018;76:488–501. 29276971 10.1016/j.chiabu.2017.12.009

[deag030-B35] Maguire-Jack K , Lanier P, Lombardi B. Investigating racial differences in clusters of adverse childhood experiences. Am J Orthopsychiatry 2020;90:106–114. 30816722 10.1037/ort0000405

[deag030-B36] Maschi T , Morgen K, Zgoba K, Courtney D, Ristow J. Trauma, stressful life events, and post traumatic stress symptoms: do subjective experiences matter? Gerontol 2011;gnr074. 10.1093/geront/gnr074 21852271

[deag030-B37] Meltzer-Brody S , Maegbaek M, Medland S, Miller W, Sullivan P, Munk-Olsen T. Obstetrical, pregnancy and socio-economic predictors for new-onset severe postpartum psychiatric disorders in primiparous women. Psychol Med 2017;47:1427–1441. 28112056 10.1017/S0033291716003020 PMC5429203

[deag030-B38] Menage J. Post-traumatic stress disorder in women who have undergone obstetric and/or gynaecological procedures: a consecutive series of 30 cases of PTSD. J Reprod Infant Psychol 1993;11:221–228.

[deag030-B39] NHS. Working definition of trauma-informed practice. 2022. https://www.gov.uk/government/publications/working-definition-of-trauma-informed-practice/working-definition-of-trauma-informed-practice (27 August 2025, date last accessed).

[deag030-B40] Nilaweera D , Phyo AZZ, Teshale AB, Htun HL, Wrigglesworth J, Gurvich C, Freak-Poli R, Ryan J. Lifetime posttraumatic stress disorder as a predictor of mortality: a systematic review and meta-analysis. BMC Psychiatry 2023;23:229. 37032341 10.1186/s12888-023-04716-w PMC10084620

[deag030-B42] Owczarek M , Karatzias T, McElroy E, Hyland P, Cloitre M, Kratzer L, Knefel M, Grandison G, Ho GWK, Morris D et al Borderline personality disorder (BPD) and complex posttraumatic stress disorder (CPTSD): a network analysis in a highly traumatized clinical sample. J Pers Disord 2023;37:112–129. 36723419 10.1521/pedi.2023.37.1.112

[deag030-B43] Redican E , Nolan E, Hyland P, Cloitre M, McBride O, Karatzias T, Murphy J, Shevlin M. A systematic literature review of factor analytic and mixture models of ICD-11 PTSD and CPTSD using the International Trauma Questionnaire. J Anxiety Disord 2021;79:102381. 33714868 10.1016/j.janxdis.2021.102381

[deag030-B44] Roozitalab S , Rahimzadeh M, Mirmajidi SR, Ataee M, Saeieh SE. The relationship between infertility, stress, and quality of life with posttraumatic stress disorder in infertile women. J Reprod Infertil 2021;22:282–288. 34987990 10.18502/jri.v22i4.7654 PMC8669410

[deag030-B45] Schein J , Houle C, Urganus A, Cloutier M, Patterson-Lomba O, Wang Y, King S, Levinson W, Guérin A, Lefebvre P et al Prevalence of post-traumatic stress disorder in the United States: a systematic literature review. Curr Med Res Opin 2021;37:2151–2161. 34498953 10.1080/03007995.2021.1978417

[deag030-B46] Shevlin M , Hyland P, Brewin CR, Cloitre M, Karatzias T, Redican E. Testing the use of “clinical checks” with the International Trauma Questionnaire to Measure PTSD and Complex PTSD. Acta Psychiatr Scand 2025;152:49–59. 40122678 10.1111/acps.13799 PMC12127064

[deag030-B47] Siqveland J , Hussain A, Lindstrøm JC, Ruud T, Hauff E. Prevalence of posttraumatic stress disorder in persons with chronic pain: a meta-analysis. Front Psychiatry 2017;8:164. 28959216 10.3389/fpsyt.2017.00164 PMC5603802

[deag030-B48] Slade P , Balling K, Sheen K, Goodfellow L, Rymer J, Spiby H, Weeks A. Work‐related post‐traumatic stress symptoms in obstetricians and gynaecologists: findings from INDIGO, a mixed‐methods study with a cross‐sectional survey and in‐depth interviews. BJOG Int J Obstet Gynaecol 2020;127:600–608. 10.1111/1471-0528.16076 31986555

[deag030-B49] Spottswood M , Davydow DS, Huang H. The prevalence of posttraumatic stress disorder in primary care: a systematic review. Harv Rev Psychiatry 2017;25:159–169. 28557811 10.1097/HRP.0000000000000136 PMC5498253

[deag030-B1] Substance Abuse and Mental Health Services Administration. *SAMHSA’s concept of trauma and guidance for a trauma-informed approach*. HHS Publication No. (SMA) 14-4884. Rockville, MD: Substance Abuse and Mental Health Services Administration, 2014.

[deag030-B50] Sunkara SK , Khalaf Y, Maheshwari A, Seed P, Coomarasamy A. Association between response to ovarian stimulation and miscarriage following IVF: an analysis of 124 351 IVF pregnancies. Hum Reprod 2014;29:1218–1224. 24651128 10.1093/humrep/deu053

[deag030-B51] Tetecher L , Cocchiaro T, Guarino A, Giannini T, Maucione S, Di Trani M, Rago R, Ciocca G. Sexological and traumatic aspects in reproductive difficulties: a psychometric study on couples seeking help for infertility. J Endocrinol Invest 2024;47:179–189. 37311972 10.1007/s40618-023-02134-z

[deag030-B52] Topp CW , Østergaard SD, Søndergaard S, Bech P. The WHO-5 Well-being index: a systematic review of the literature. Psychother Psychosom 2015;84:167–176. 25831962 10.1159/000376585

[deag030-B53] Verhaak CM , Smeenk JM, Evers AWM, Kremer JM, Kraaimaat FW, Braat DM. Women’s emotional adjustment to IVF: a systematic review of 25 years of research. Hum Reprod Update 2007;13:27–36. 16940360 10.1093/humupd/dml040

[deag030-B54] Wang Y , Fu Y, Ghazi P, Gao Q, Tian T, Kong F, Zhan S, Liu C, Bloom DE, Qiao J. Prevalence of intimate partner violence against infertile women in low-income and middle-income countries: a systematic review and meta-analysis. Lancet Glob Health 2022;10:e820–e830. 35561719 10.1016/S2214-109X(22)00098-5 PMC9115867

[deag030-B55] WHO. ICD-11: International Classification of Diseases, 11th revision edn, 2022.

[deag030-B56] Yang SR , Yeo JH. Effects of irrational parenthood cognition, post traumatic stress disorder and spousal support on quality of life of infertile women. Korean J Women Health Nurs 2017;23:145–153. 37684894 10.4069/kjwhn.2017.23.2.145

