# Supplementary material for: Experiences of infertility-related traumatic events and their association with symptoms of Post-Traumatic Stress Disorder (PTSD) and Complex PTSD: results from a mixed-methods online survey
Source: Hum Reprod. 2026 Mar 12;41(5):772–85. doi: 10.1093/humrep/deag030 (PMC13139654; doi:10.1093/humrep/deag030)
Supplement: deag030_Supplementary_Figure_S1 [file deag030_supplementary_figure_s1.pdf]

Human Reproduction, 2026, 1–1
https://doi.org/10.1093/humrep/deag030
Supplementary Data

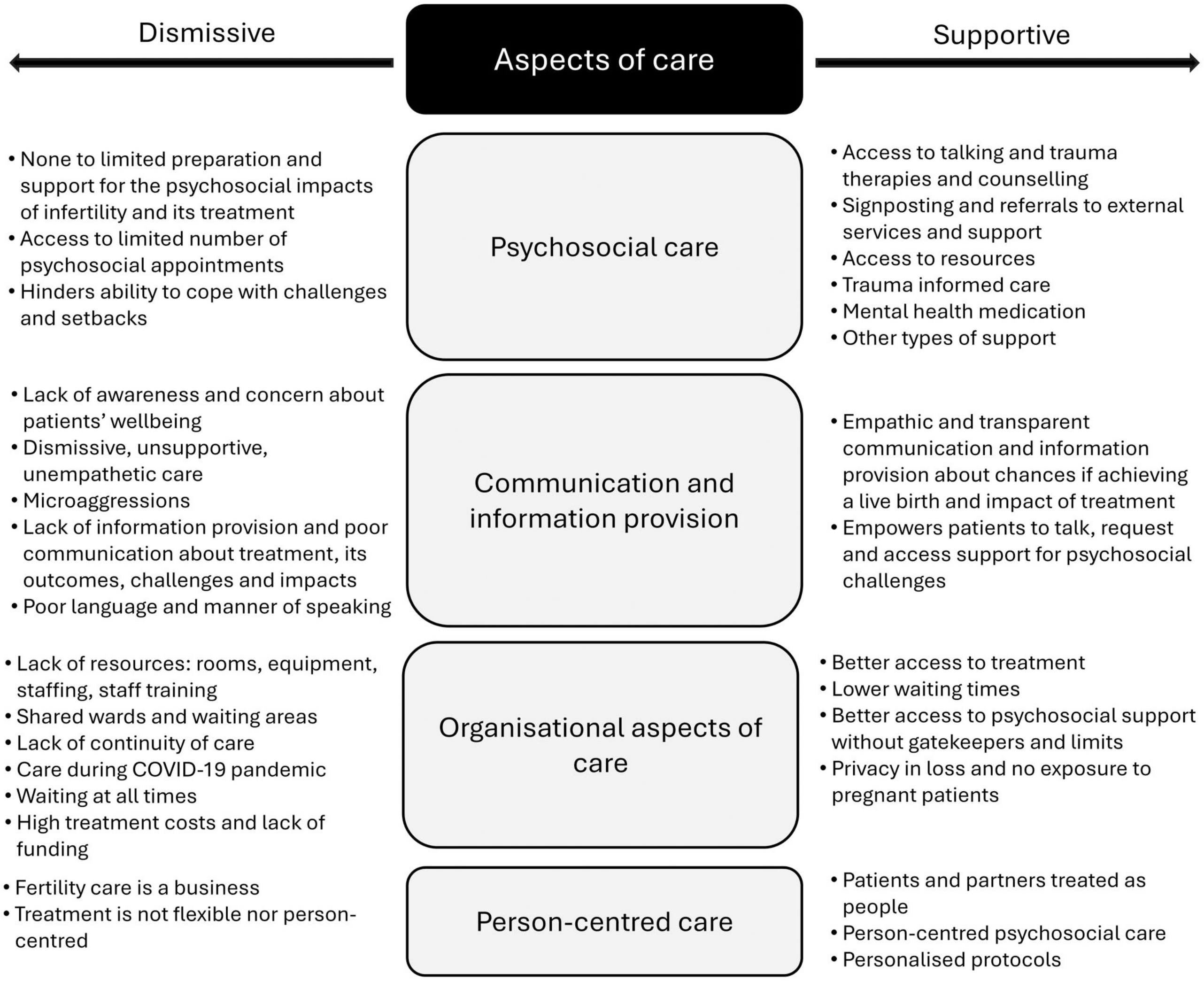

Supplementary Figure S1. Thematic map representing the four meta-themes (in grey) identified in qualitative analysis of reproductive and infertility-related care (N = 590). For detailed descriptions of each theme and their categories, prevalence and level of endorsement by participants, and illustrative quotes refer to Supplementary Tables S8, S9, S10, and S11.
