# Supplementary material for: Experiences of infertility-related traumatic events and their association with symptoms of Post-Traumatic Stress Disorder (PTSD) and Complex PTSD: results from a mixed-methods online survey
Source: Hum Reprod. 2026 Mar 12;41(5):772–85. doi: 10.1093/humrep/deag030 (PMC13139654; doi:10.1093/humrep/deag030)
Supplement: deag030_Supplementary_Table_S1 [file deag030_supplementary_table_s1.pdf]

OXFORD
human reproduction

Human Reproduction, 2026, 1–1
https://doi.org/10.1093/humrep/deag030
Supplementary Data

Supplementary Table S1. Qualitative theme *Fertility journey is extremely distressful*, its categories, number of codes (*k*), and proportion (%) of total codes.

Theme and categories description	Total sample *k* (%)/1714 codes	Illustrative quotes
**Theme: Fertility journey is extremely distressful**<br>Fertility journey is an extremely distressful experience due to the complex interplay of several stressful factors that patients routinely endure.<br>**Categories are:**	378 (22%)	
**Treatment is emotionally taxing**<br>Fertility treatment is a challenging experience, involving a rollercoaster of emotions, with heartbreak, hope, loss and anxiety affecting patients' wellbeing and mental health in extreme ways.	179 (10%)	'I spiralled emotionally and lashed out at people close to me in my life, and started drinking alcohol to numb the pain, which felt melodramatic, but I just felt so depressed and alone, and that there had been no duty of care shown to me'. P 312, Did not meet criteria for (C)PTSD<br>'The balancing of appointments, work, friendships, relationships whilst undergoing emotional stress, physical/hormonal changes/moods and the on-going hope/grief cycle'. P 57, Met criteria for (C)PTSD<br>'My ICSI resulted in pregnancy and then we lost the baby having been diagnosed with a miscarriage at 3 months. The way I was treated during my miscarriage was appalling and resulted in PTSD for my husband'. P 23, Did not meet criteria for (C)PTSD
**Treatment has cumulative negative effects**<br>IVF cycles have a cumulative physical and emotional toll that can result in decline in physical and mental health. Fertility treatment has multiple side-effects, which can be impairing.	101 (5%)	'I'm not sure there's one experience and perhaps the question itself illustrates at least a degree of lack of insight into infertility as the effects can be accumulative and can impact even quite unconsciously over time'. P 201, Met criteria for (C)PTSD<br>'Things got harder with each failed attempt. I hid what we were going through [treatment] at work so had to keep functioning through the cycles'. P 204, Did not meet criteria for (C)PTSD<br>'Post egg collection pelvic infection and OHSS resulted in two weeks stay in hospital needing emergency surgery and signing a consent form with possibility of hysterectomy'. P 104, Met criteria for (C)PTSD<br>'I've also experienced skin complaints and migraines since failed IVF so I'm not sure what other health issues await me'. P 280, Met criteria for (C)PTSD
**Fertility treatment is all consuming**<br>Fertility journey often takes over patients' lives due to financial, emotional and physical demands of the treatments.	30 (2%)	'I felt consumed by infertility. I felt like everyone was moving on with their lives and I was stuck. I stopped socialising I stopped meeting friends. I didn't want to hear about my friends' lives. My in-laws stopped speaking with me as they didn't agree with fertility treatment. I stopped going for out of dinner in case this affected my eggs. I was upset with my husband if he had a glass of wine'. P 464, Met criteria for (C)PTSD<br>'It's hard because no one understands the depth of the suffering caused by infertility. It affects every part of my life—my relationship with my husband, my sex life, my finances, my ability to focus on work, my mental health, my physical health and my friendships'. P 92, Did not meet criteria for (C)PTSD
**Suffering due to unfulfilled child desire**<br>Contrast between the extreme desire and investment put into having children and the treatment outcome results in devastating disillusionment and lack of meaning in life	28 (2%)	'To put my body through all the injections, medications and scans for it to be unsuccessful was devastating. I felt like a failure. I was angry at the clinic and angry at all my friends who got pregnant naturally. This failed round was only a few months ago and I still feel angry, depressed and hopeless'. P 73, Did not meet criteria for (C)PTSD<br>'Daily sadness that comes with not being able to have children'. P 404, Met criteria for (C)PTSD
**Invasive nature of fertility treatment**<br>The repeated intrusive and mechanistic treatment of the patient body in treatment procedures, resulting in exhaustion, fatigue, depletion.	27 (2%)	'The constant intrusive tests for women, constantly having speculums put in you, constant tests and being made to feel like it's your fault and being told you are fat a lot!' P 436, Did not meet criteria for (C)PTSD<br>'I am so emotionally drained from being pulled and poked around and tested with bloods and weight etc. I mentally don't think I have the fight in me to try get my treatment'. P 279, Met criteria for (C)PTSD
**There is no joy in MAR pregnancy**<br>Despite the initial excitement of successful MAR, pregnancy is often experienced as anxiety provoking due to fears that it might go wrong and previous negative fertility treatment experiences.	13 (1%)	'Even being pregnant is not joyful due to the fear of it all being taken away. The joy of a surprise pregnancy test, the joy of announcements to family, the joy of being blissfully ignorant to what could go wrong and just enjoying pregnancy and telling the world and buying all the things. Instead of joy you have anxiety and fear'. P 26, Met criteria for (C)PTSD
