# Supplementary material for: Experiences of infertility-related traumatic events and their association with symptoms of Post-Traumatic Stress Disorder (PTSD) and Complex PTSD: results from a mixed-methods online survey
Source: Hum Reprod. 2026 Mar 12;41(5):772–85. doi: 10.1093/humrep/deag030 (PMC13139654; doi:10.1093/humrep/deag030)
Supplement: deag030_Supplementary_Table_S2 [file deag030_supplementary_table_s2.pdf]

OXFORD
human reproduction
Human Reproduction, 2026, 1–1
https://doi.org/10.1093/humrep/deag030
Supplementary Data

Supplementary Table S2. Qualitative theme *Dismissive care*, its categories, number of codes (*k*), and proportion (%) of total codes.

Theme and categories description	Total sample *k* (%)/1714 codes	Illustrative quotes
**Theme: Dismissive care** Fertility healthcare in which patients' concerns and symptoms are not taken seriously by the fertility treatment providers, involving perceptions of disrespect, inconsideration, rudeness, resulting in suboptimal treatment, increased anxiety and patient dissatisfaction.	364 (21%)	
**Categories are:**		
**Lack of empathy and compassion** Perceptions of being treated in an insensitive way and without compassion by healthcare professionals	146 (8%)	'Dealing with the clinics was extremely stressful until we finally found one that focussed on patient care for the last transfer. Clinics in the UK don't tend to put patients first. And they certainly don't tend to employ best practice when treatment doesn't work in that they don't seek to provide any support. It made the experience a lot more stressful than it needed to be dealing with lack of empathy'. P 49, Did not meet criteria for (C)PTSD
**Poor communication and information provision at all stages of treatment** Patients reported there was limited communication and preparation before, during and after IUI and IVF cycles, the lack of transparency about treatment success rate and poor information sharing with patients.	110 (6%)	'Having a HyCoSy scan was the most traumatic part of my fertility experience. I had very little pre information about what to expect, was instructed quite abruptly that I had to take a pregnancy test at the beginning of the scan (upsetting and a clear lack of reading of my notes by doctors) and then found the scan very painful. I was made to feel like I shouldn't have found it painful'. P 573, Did not meet criteria for (C)PTSD<br>'Communication throughout was appalling .... misinformation on medication, procedures etc'. P 289, Met criteria for (C)PTSD<br>'I understand the NHS is under pressure and this is not seen as essential, but being belittled, misled and dismissed during such an emotive time makes you feel more desperate and alone'. P 93, Met criteria for (C)PTSD
**Lack of psychosocial support** Clinics are seen to provide very limited guidance and support for the psychosocial impacts of infertility and its treatment (i.e. counselling, psychosocial care), resulting in patients' overwhelm and negatively affecting their ability to cope with treatment setbacks and challenges.	66 (4%)	'Transfer failed. No support given. No offer of counselling. We were devastated after each failed round and clinic offered absolutely no support'. P 19, Met criteria for (C)PTSD<br>'I had little to no support for my issues as a male. Ex wife left the marriage's and considered suicide due to my infertility issues'. P 3, Did not meet criteria for (C)PTSD
**Structural barriers to empathetic care** NHS and private clinics' resources (waiting areas, rooms, equipment, staffing, staff training) and logistical limitations (lack of continuity of care, shared resources (rooms, walkways and staff) with ob-gyn and maternity wards compound the feelings of poor care and lack of compassion towards patients who end treatment without children.	27 (2%)	'I had to wait in a room with a lot of pregnant people and explain everything about my first exec to the doctor. There was no heartbeat or sac on the ultrasound. Following this I then had to visit the general Obstetrics and Gynaecology ward for a scan for them to confirm what I had just been told as the information could not be shared from the clinic. I was seated in a waiting room with women with visible bumps happily awaiting their regular monitoring scans. It was beyond heartbreaking'. P 432, Met criteria for (C)PTSD<br>'And no end-to-end care with the same health provider. Once I was pregnant, clinic send you to regular clinics to await scans. I don't believe they account for IVF women maybe needing more attention/ having more anxiety'. P 438, Met criteria for (C)PTSD
**Fertility care is a business** Private sector fertility clinics are seen as a part of a growing industry where profit is made at the cost of patients' experience, their wellbeing and health.	15 (1%)	'Also, the insensitivity of feeling like I was on a conveyer belt as so fast .... Very money orientated and doctors and staff act like God as creating (or not in my case) children'. P 99, Did not meet criteria for (C)PTSD<br>'Being led on a merry go round of hope, being offered treatment after treatment. No consideration to the emotional harm. Sold additional treatments based on hope. I'm sure my consultant fully knew that I would never conceive'. P 47, Met criteria for (C)PTSD
