# Supplementary material for: Experiences of infertility-related traumatic events and their association with symptoms of Post-Traumatic Stress Disorder (PTSD) and Complex PTSD: results from a mixed-methods online survey
Source: Hum Reprod. 2026 Mar 12;41(5):772–85. doi: 10.1093/humrep/deag030 (PMC13139654; doi:10.1093/humrep/deag030)
Supplement: deag030_Supplementary_Table_S3 [file deag030_supplementary_table_s3.pdf]

OXFORD
human reproduction

Human Reproduction, 2026, 1–1
https://doi.org/10.1093/humrep/deag030
Supplementary Data

Supplementary Table S3. Qualitative theme *Lack of control*, its categories, number of codes (*k*), and proportion (%) of total codes.

Theme and categories description	Total sample *k* (%)/1714 codes	Illustrative quotes
**Theme:** **Lack of control** Fertility care is characterized by an experience of severely limited control over several aspects of the treatment. **Categories are:**	270 (16%)	
**Structural factors** The high treatment cost incurs potential debt due to very limited NHS support, with access varying according to the geolocation and individual circumstances. People with secondary infertility and same sex couples are not eligible for funding, resulting in financial strain and a sense of unfairness.	82 (5%)	'Gay tax making you feel like 3rd class citizen'. P155, Met criteria for CPTSD<br>'I feel IVF is extremely expensive for working people and we still have debts to pay with no baby. We had 4 ICSI cycles privately and think the whole thing is a scam with very little chance of success for a high price'. P 196, Met criteria for CPTSD<br>'Secondly, the postcode lottery of NHS funding is really awful, it feels really unfair that someone who lives minutes away from you can have more funded cycles than you'. P 366, Met criteria for CPTSD
**Uncertainty of treatment outcome** The lack of guarantee whether the treatment will work and/or when it will work despite the willingness to sacrifice a lot.	81 (5%)	'The uncertainty of if it would happen for us was the most traumatic element because I couldn't control that'. P 424, Did not meet criteria for (C)PTSD<br>'It was the thought of being childless and the no guarantees of treatment being successful despite many attempts and the financial implications'. P 8, Did not meet criteria for (C)PTSD
**Waiting** Having to wait at all levels of fertility treatment and experiencing delays in treatment, also due to the Covid-19 pandemic.	85 (5%)	'The waiting between each part of stimulating ovaries scans and then seeing if it had fertilised and then actually worked. Then if it hadn't worked, waiting to hear the plan. Also waiting to see if you miscarry … '. P 99, Did not meet criteria for (C)PTSD<br>'It took 2 years from NHS beginning tests to then finally being referred for IVF. This was following the 12 months we had been recommended to keep trying naturally. We spent up to 3 months just waiting for routine appointments such as blood tests etc. We then had our long-awaited cycle cancelled at the start of Covid with no explanation as to when we could begin in the future'. P 310, Did not meet criteria for (C)PTSD
**Treatment is not flexible nor person-centred** Patients perceive treatment is individualized. They perceive a lack of options and opportunity to get second opinions, leading to frustration and disappointment.	22 (1%)	'How impersonal it is, but you are part of a factory and your faulty and if the standard thing doesn't work you are tossed away'. P 223, Met criteria for CPTSD<br>'I emailed my clinic to inform them of my negative test on the day and a nurse replied on the next working day (it was a weekend) to say she was sorry to hear that and that counselling was available. I replied by saying I just want to talk to my doctor about what we can learn from the failed round. I didn't hear back for several days, and I chased and chased. I felt unsupported and ignored, and it made the trauma of having my hopes dashed worse. To feel alone and like the doctor didn't care at all. I was just a number to her'. P 554, Did not meet criteria for (C)PTSD
