# Supplementary material for: Experiences of infertility-related traumatic events and their association with symptoms of Post-Traumatic Stress Disorder (PTSD) and Complex PTSD: results from a mixed-methods online survey
Source: Hum Reprod. 2026 Mar 12;41(5):772–85. doi: 10.1093/humrep/deag030 (PMC13139654; doi:10.1093/humrep/deag030)
Supplement: deag030_Supplementary_Table_S4 [file deag030_supplementary_table_s4.pdf]

Human Reproduction, 2026, 1–1
https://doi.org/10.1093/humrep/deag030
Supplementary Data

Supplementary Table S4. Qualitative theme *Reproductive loss*, its categories, number of codes (*k*), and proportion (%) of total codes.

Theme and categories description	Total sample *k* (%)/1714 codes	Illustrative quotes
**Theme: Reproductive loss** The experience of not being able to have a child or the loss of a pregnancy at any stage, including miscarriage, stillbirth, and infertility. It encompasses a broad range of events impacting family formation and can result in significant emotional and psychological distress.	269 (16%)	
**Categories are:**		
**Embryo loss, miscarriage and baby loss** Many people undergoing fertility treatment experience embryo and pregnancy loss including miscarriages (early pregnancy loss), stillbirths (late pregnancy loss) and perinatal deaths (death of a baby within the first weeks of life).	149 (9%)	'[When my embryos didn't fertilize properly during IVF it felt like I had lost my babies—that they had died'. P 425, Met criteria for (C)PTSD<br>'Having a miscarriage 6 weeks into my first and only pregnancy and not being able to conceive afterwards (…)'. P 168, Did not meet criteria for (C)PTSD
**Shock of not being able to conceive** The realization of the inability to conceive and/or diagnosis of subfertility or infertility is perceived as unexpected and shocking.	68 (4%)	'I am a man that was diagnosed with [rare condition] in 2012 following unsuccessful attempts to conceive with my wife. A complete shock to find out that I was [and always had been] infertile'. P 469, Did not meet criteria for (C)PTSD<br>'I found that initially getting our diagnosis was so troubling and led to depression and anxiety for me. It took a long time before I could accept our position and tackle it with a positive mindset. That for me was definitely the most difficult part. You are told in a 30 min consult all this info that is just mind blowing'. P 469, Did not meet criteria for (C)PTSD
**Unacknowledged secondary infertility** The inability to conceive or carry a pregnancy to term after previously having a child is often unacknowledged due to societal perceptions, resulting in lesser fertility treatment support and limited understanding for secondary infertile patients.	34 (2%)	'Having to bear the weight of infertility whilst being in the position of already having a child has also caused a lot of issues- your feelings are normally completely invalidated by professionals because you've been fortunate to conceive previously'. P 210, Met criteria for (C)PTSD<br>'We had secondary infertility and the views of other people whether that was that we were selfish for not having another child or we should be grateful for having one. I struggled with friends that had multiple children easily but also with friends that hadn't managed to have one child. We were somewhere in the middle'. P 91, Did not meet criteria for (C)PTSD
**Grief is a part of fertility treatment and loss is unacknowledged** Grieving each reproductive loss is an integral and commonly experienced part of fertility journey. It's intensity and longevity are unacknowledged, unrecognized. Participants frame it as an invisible loss of future.	18 (1%)	'I don't want to compare grief. But people generally understand miscarriages and the potential for grief and loss. But "just" not getting pregnant is not really recognised as another type of grief … However, for me each month felt like a type of miscarriage … I would commit myself fully to becoming a mum. And each month I would fail. And each month a small piece of me, or my sanity, hope and peace would fail too. Death by a thousand cuts … '. P 217, Did not meet criteria for (C)PTSD<br>'Everything about it is traumatizing—physical violation, constant emotional trauma, (…), the absolute utter grief of it. Grief that has nowhere really to go because it's a grief for something that never was.' P 590, Did not meet criteria for (C)PTSD
