# Supplementary material for: Experiences of infertility-related traumatic events and their association with symptoms of Post-Traumatic Stress Disorder (PTSD) and Complex PTSD: results from a mixed-methods online survey
Source: Hum Reprod. 2026 Mar 12;41(5):772–85. doi: 10.1093/humrep/deag030 (PMC13139654; doi:10.1093/humrep/deag030)
Supplement: deag030_Supplementary_Table_S5 [file deag030_supplementary_table_s5.pdf]

OXFORD
human reproduction
Human Reproduction, 2026, 1–1
https://doi.org/10.1093/humrep/deag030
Supplementary Data

Supplementary Table S5. Qualitative theme *Social impact*, its categories, number of codes (k), and proportion (%) of total codes.

Theme and categories description	Total sample *k* (%)/1714 codes	Illustrative quotes
**Theme:** **Social impact** Fertility journey presents significant challenges and strains to all kinds of relationships and work.	231 (13%)	
**Categories are:** **Suffering alone** Fertility journey is isolating due to emotional burden experienced, difficulty in sharing the experience, social perceptions, cultural taboos, and lack of support.	75 (4%)	'If you are not living it, even those closest to you don't understand. It's lonely to be in the dark with only your thoughts—it's terrifying'. P 263, Did not meet criteria for (C)PTSD<br>'Feeling like it was only us that it was happening to and being completely uneducated on what fertility treatment involved'. P 163, Met criteria for (C)PTSD
**Impact on partnership** Fertility journey can result in increased stress, strained communication, emotional challenges, intimacy problems, and financial cost for the couple.	46 (3%)	'I struggle with dealing with the fact that it is me that has the diagnosis and not my husband. My husband could create a family with anyone, as he wants a family so bad and I cannot seem to give him that, when he deserves it so much'. P 400, Met criteria for (C)PTSD<br>'Terrible effect on own and partners mental health. Drove us apart. And part of reason for divorce'. P 27, Met criteria for (C)PTSD<br>'The remaining unresolved trauma, I am unable to be intimate with my husband because I felt so violated by all the procedures and surgeries needed over many years. The fear of creating a less-than-ideal environment for the embryo meant intercourse felt bad and risky. You are even told to abstain which doesn't help'. P 235, Did not meet criteria for (C)PTSD
**Jealousy and anger towards friends and family** Friends' pregnancies and baby news when experiencing own infertility, undergoing fertility treatment, or experiencing failed IFV cycles bring a sense of injustice and difficult feelings of jealousy, envy and anger.	49 (3%)	'I still feel pangs of jealousy when friends tell me they're pregnant—that feeling never seems to go away'. P 202, Did not meet criteria for (C)PTSD<br>'The hardest thing was my husband already having a child (then having male factor infertility when we started trying) and witnessing all his milestones not knowing if I would have that with my own child. The pain of sitting through family gatherings with his family commenting on how alike they were and jokes about how my husband might be a grandad soon plus various other insensitive comments was very isolating'. P 242, Met criteria for (C)PTSD
**Fertility treatment negatively impacts work** Difficulties at work during or after treatment. Treatment and its side effects interfere with productivity, and patients perceive a lack of understanding from workplace managers. Some workplaces were seen as stressful (e.g. schools, midwifery and antenatal care) due to their nature of working with babies, children and parents, and general stress.	34 (2%)	'Support at work is minimal and people don't understand the difficulty of infertility. It is a medical procedure, and you should be off work during a cycle'. P 196, Met criteria for (C)PTSD<br>'I felt utterly bereft and had no idea what to do next. I ended up leaving my job as a teacher because I could no longer cope with managing such a stressful career alongside the grief that came with the loss of those final embryos and, what I believed at the time, was the end of my fertility journey. It was a double loss. The loss of my embryos, and the loss of a career I loved and had dedicated my life to'. P 298, Did not meet criteria for (C)PTSD
**Lack of support and understanding from friends and family** People affected by infertility can struggle with perceptions of lack of support from their family feeling dismissed and misunderstood by their friends and family.	27 (1%)	'It's very difficult every time a friend or family announces a pregnancy and the questions that then come your way about why you haven't started a family. The thought of the questions causes me great anxiety'. P 582, Did not meet criteria for (C)PTSD<br>'The hardest part is trying to navigate a whole change in your world and people close to you not really understanding how hard it is emotionally'. P 377, Did not meet criteria for (C)PTSD
