# Supplementary material for: Experiences of infertility-related traumatic events and their association with symptoms of Post-Traumatic Stress Disorder (PTSD) and Complex PTSD: results from a mixed-methods online survey
Source: Hum Reprod. 2026 Mar 12;41(5):772–85. doi: 10.1093/humrep/deag030 (PMC13139654; doi:10.1093/humrep/deag030)
Supplement: deag030_Supplementary_Table_S6 [file deag030_supplementary_table_s6.pdf]

OXFORD
human reproduction

Human Reproduction, 2026, 1–1
https://doi.org/10.1093/humrep/deag030
Supplementary Data

Supplementary Table S6. Qualitative theme *Medical trauma*, its categories, number of codes (*k*), and proportion (%) of total codes.

Theme and categories description	Total sample *k* (%)/1714 codes	Illustrative quotes
**Theme:** **Medical trauma** Physiological and psychological responses that individuals experience in reaction to fertility treatment diagnosis, procedures, events, and outcomes, leading to distress. **Categories are:**	120 (7%)	
**Painful and traumatic procedures and surgeries** Infertility treatments involve painful procedures like egg retrieval and hysteroscopy. People can also experience traumatic surgeries because of ectopic pregnancies and miscarriages. Patients perceive their pain and distress reactions are dismissed.	74 (4%)	'I was traumatised by the egg retrieval. The clinic only has analgesia, not sedation, and it was so sore, so traumatising and I imagine it was not easy to do on me as I was writhing in pain. I was sore for days and tender and remained [in] very low mood'. P 247, Did not meet criteria for (C)PTSD<br>'The doctor performing the transfer was new and after 15 mins of trying to insert the embryo asked another member of staff to try. This continued until eventually the consultant was brought in and I was given gas and air'. P 475, Met criteria for (C)PTSD
**Mistakes in diagnosis and treatment** Patients perceived errors in infertility diagnosis and treatment resulted in delayed and/or inappropriate interventions, with a potential to decrease chances of conception and impact reproductive health.	36 (2%)	'The majority of the trauma has been caused by human error and miscommunication'. P 114, Met criteria for (C)PTSD<br>'Being told after two failed rounds of ICSI that I would not conceive with own eggs when the diagnosed issue with our fertility was husband's sperm, which clinic refused to look into'. P 262, Did not meet criteria for (C)PTSD<br>'I found out at my second fertility specialist that my first infertility specialist had been using procedures for me that were meant for patients using fresh not frozen sperm. The doctor was not having me contact them when I was ovulating, based on me testing. They chose a certain point in my cycle, and I would go to the office and have an internal exam for them to determine if the proceeding day or several days was appropriate. Based on ovulation testing at my second clinic the dates of my inseminations were all too early for conception to have occurred'. P 16, Met criteria for (C)PTSD
**Long-term health conditions affecting and affected by treatment** Several long-term health conditions impact fertility and/or can interact with fertility treatment. Finding about unexpected new disease often ads complexity to treatment, and treatment can result in flare ups of other diseases.	10 (1%)	'Having to choose to have chemotherapy over the chance of having children'. P 404, Met criteria for (C)PTSD<br>'I had IVF (ICSI), and it failed before transplant. As a result of the hormonal treatments my endometriosis flared up and led me to the decision to stop fertility treatments. I had to go to A&E because of the pain, thinking it was OHSS, but it was pain caused by endometriosis'. P 143, Did not meet criteria for (C)PTSD<br>'Additionally, I felt enormous guilt and stress about managing blood sugars, which would cause me anxiety when they were outside of target, despite doing well at managing the condition. The diabetes team give target glucose levels but offer no guidance on how to achieve them. I found out from a consultant that patients often don't achieve these aspirational targets. Giving an unachievable target to a patient undergoing IVF treatment with unexplained infertility caused me more harm than good'. P 396, Met criteria for (C)PTSD
