# Supplementary material for: Experiences of infertility-related traumatic events and their association with symptoms of Post-Traumatic Stress Disorder (PTSD) and Complex PTSD: results from a mixed-methods online survey
Source: Hum Reprod. 2026 Mar 12;41(5):772–85. doi: 10.1093/humrep/deag030 (PMC13139654; doi:10.1093/humrep/deag030)
Supplement: deag030_Supplementary_Table_S7 [file deag030_supplementary_table_s7.pdf]

OXFORD
human reproduction
Human Reproduction, 2026, 1–1
https://doi.org/10.1093/humrep/deag030
Supplementary Data

Supplementary Table S7. Qualitative theme *Broken Self-Identity*, its categories, number of codes (k), and proportion (%) of total codes.

Theme and categories description	Total sample *k* (%)/1714 codes	Illustrative quotes
**Theme:** **Broken Self-Identity** The need for fertility treatment and the experience of infertility result in a broken sense of self, self-blame, and negative feelings towards oneself and own body.	92 (5%)	
**Categories are:** **Failing to meet gender societal expectations** Infertility and the need for fertility treatment are experienced as stigma with potential to damage social standing and support, relationships, cause anxiety and depression, decrease self-esteem and self-efficacy	48 (3%)	'I felt that if I was out of the way he could meet someone else and have a family with someone who wasn't as flawed as me and was holding him back from a lifetime of being a father'. P 70, Met criteria for (C)PTSD<br>'Not being thought of as a woman. Along with being perceived as non-existent'. P 214, Met criteria for (C)PTSD<br>'I suffer with severe anxiety and depression now as I still cannot provide my husband with a child'. P 279, Met criteria for (C)PTSD<br>'Feeling like a failure and not living up to people's expectations that we should have children by now'. P 500, Met criteria for (C)PTSD
**Depersonalisation and body dysphoria** A feeling of detachment and disconnection from one-self and one's body because of fertility treatment. A feeling of hatred towards one's body because of not being able to conceive naturally and going through unsuccessful fertility treatments.	25 (1%)	'It feels like my body is not my own and everybody has seen it/prodded it and, although I am proud of it for producing my miracle baby, it doesn't feel like my own anymore as it has changed so much'. P 572, Did not meet criteria for (C)PTSD<br>'Putting so much hope, putting your body through so much just to fail repeatedly. losing your identity, changes to your body, not recognising yourself. becoming obsessed, preoccupied, desperate'. P 101, Met criteria for (C)PTSD<br>'The news it hadn't worked again was indescribable. I have never felt that desperate. I screamed for about half an hour and had to be calmed down. These experiences have caused me to truly hate my own body. I've never felt so angry, and it is all anger towards myself'. P 54, Met criteria for (C)PTSD<br>'The frustration at my own body for not doing what it's expected of it'. P 61, Did not meet criteria for (C)PTSD
**Guilt and shame** Fertility patients experience psychological distress in feelings of guilt and shame about their inability to have children. The idea that fertility is their responsibility feeds the self-blame for their mind and bodies not working as they are meant to.	19 (1%)	'The predatory nature of the fertility "market" - supplements, holistic treatments, diets etc. - the things that make you feel like infertility is your fault for the things you have or haven't done'. P 581, Did not meet criteria for (C)PTSD<br>'The worst part of the IVF was when it failed. It wasn't the physical part of this, it was emotional. I felt like it was my fault as l had been so anxious. I felt like l had caused it not to work because of something l had done. This stayed with me for a long time. l felt a lot of guilt that if l had been more positive l would have been pregnant'. P 118, Did not meet criteria for (C)PTSD
