# Supplementary material for: Experiences of infertility-related traumatic events and their association with symptoms of Post-Traumatic Stress Disorder (PTSD) and Complex PTSD: results from a mixed-methods online survey
Source: Hum Reprod. 2026 Mar 12;41(5):772–85. doi: 10.1093/humrep/deag030 (PMC13139654; doi:10.1093/humrep/deag030)
Supplement: deag030_Supplementary_Table_S8 [file deag030_supplementary_table_s8.pdf]

OXFORD
human reproduction
Human Reproduction, 2026, 1–1
https://doi.org/10.1093/humrep/deag030
Supplementary Data

Supplementary Table S8. Qualitative theme *Positive Experiences of care*, its categories, number of codes (*k*), and proportion (%) of total codes.

Theme and categories description	Total sample *k* (%)/250	Illustrative quotes
**Theme:**<br>**Positive experiences of reproductive or fertility care**<br>Experiences of Care received from healthcare professionals that helped to cope with fertility treatment related trauma.<br>**Categories are:**	250 (100%)	
**Undergoing psychological therapy**<br>Having received and/or undergoing talking therapy, including psychology sessions, Cognitive-Behavioural Therapy, EMDR, IAPT, hypnotherapy and mindfulness. Most of sessions provided by fertility counselling services and often limited to one, three or six sessions.	127 (51%)	'Offered 2 x fertility counselling appointments per cycle—I have accessed this twice since the start of my treatment'. P 586, Did not meet criteria for (C)PTSD<br>'There was a counsellor at the fertility centre, but support was one off and minimal. Made me realise I needed further support though which was good'. P 120, Did not meet criteria for (C)PTSD<br>'[I had] EMDR therapy for 8 weeks'. P 506, Met criteria for (C)PTSD<br>'[I]Have accessed IAPT. Put on waiting list for CBT'. P 364, Met criteria for (C)PTSD<br>'[I have received] talking therapy & hypnotherapy'. P 201, Met criteria for (C)PTSD<br>'(…) I accepted the free counselling sessions (I believe it was 2 or 3)'. P 228, Met criteria for (C)PTSD
**Receiving offers of counselling**<br>When the healthcare professionals discussed how infertility and its treatment might affect participants, they offered counselling or suggested referring to counselling services.	65 (26%)	'Offered 3 counselling session per ICSI cycle'. P 435, Did not meet criteria for (C)PTSD<br>'Offered counselling from the fertility clinic when we had to abandon the first cycle'. P 564, Did not meet criteria for (C)PTSD<br>'We were informed that it could be difficult and offered free counselling and support groups'. P 567, Did not meet criteria for (C)PTSD
**Discussions regarding fertility patients' expectations of fertility journey, and the feelings and emotions in their fertility journey.**<br>Having the opportunities to talk about difficult experiences, reproductive loss, emotional impact of treatment, treatment success statistics, feelings towards future children, debriefing after treatments phases, and other fertility-related topics.	26 (10%)	'We spoke about trying to conceive'. P 198, Did not meet criteria for (C)PTSD<br>'A doctor attached to a private health clinic I attend said to me that unfortunately they can't do anything to help me with the emotional side of things—he was realistic about what his clinic had to offer, and I appreciated that he really validated just how real and difficult what I am experiencing is. This was very touching and meaningful for me to hear from a professional'. P 495, Met criteria for (C)PTSD<br>'(…) and also consultants have been good at describing what may happen and then debriefing afterwards'. P454, Did not meet criteria for (C)PTSD
**Signposting and referrals to external services**<br>Being signposted to support groups, Fertility Network, other external help resources, perinatal mental health services and other services.	14 (6%)	'Referred to miscarriage support group'. P 374, Did not meet criteria for (C)PTSD<br>'They provided a list of fertility therapists, support group access and webinars'. P 370<br>'Signposting to Fertility Network UK'. P 564, Did not meet criteria for (C)PTSD
**Other support**<br>Receiving other types of support, unspecified help, supportive contact after reproductive loss, psychiatric evaluation, memory box offered.	10 (4%)	'They offered me a memory box and offered to phone me a week after the D&C for my miscarriage'. P 367, Did not meet criteria for (C)PTSD
**Mental Health Medication**<br>Being offered or prescribed medication for anxiety and/or depression.	8 (3%)	'Aforementioned perinatal mental health service—had talking therapy and sertraline prescription postpartum'. P 121, Met criteria for (C)PTSD<br>'I started on antidepressants through GP after we gave up on treatment with my own eggs and while my husband said he would not progress with egg donation, adoption or fostering. I was at a crossroads where I felt I had to choose between my marriage and having children'. P 389, Did not meet criteria for (C)PTSD
