# Supplementary material for: Experiences of infertility-related traumatic events and their association with symptoms of Post-Traumatic Stress Disorder (PTSD) and Complex PTSD: results from a mixed-methods online survey
Source: Hum Reprod. 2026 Mar 12;41(5):772–85. doi: 10.1093/humrep/deag030 (PMC13139654; doi:10.1093/humrep/deag030)
Supplement: deag030_Supplementary_Table_S9 [file deag030_supplementary_table_s9.pdf]

OXFORD
human reproduction

Human Reproduction, 2026, 1–2
https://doi.org/10.1093/humrep/deag030
Supplementary Data

Supplementary Table S9. Qualitative theme *Dismissive Care*, its categories, number of codes (k), and proportion (%) of total codes.

Themes and categories description	Total sample *k* (%) /415 codes	Illustrative quotes
**Theme** **Dismissive care** Fertility healthcare in which patients' concerns and symptoms are not taken seriously by healthcare providers, involving experiences of disrespect, inconsideration, rudeness, resulting in suboptimal treatment, increased anxiety and patient dissatisfaction.	348 (84 %)	
**Categories are:**		
**Attitudes and personal attributes of medical and healthcare professionals** Healthcare professionals expressing a lack of awareness or concern for the feelings, needs or circumstances of others, disregarding or minimizing the impact of their words or actions on fertility patients' emotional wellbeing.	67 (16%)	'On one cycle, I was brought to theatre late for egg collection and it transpired that I had already ovulated. The medical director was rude and obnoxious and suggested to us that if we got a refund that we would have to destroy the one embryo that made it to blastocyst stage. For my next FET, this horrible person was the only doctor available to do a [clinical] procedure on me. It was so degrading. I felt like taking my foot out of the stirrup and kicking him away from me. I cried the entire time'. P 114, Met criteria for (C)PTSD<br>'The clinic seemed to just shrug their shoulders time and time again when things "didn't go to plan" and didn't seem to want to work out why, then when we asked what more we could do it was always "oh well we could try this thing" and the next time "oh there's this treatment" when they could have suggested it all at the beginning and we could decide if we wanted to pursue those things instead of having doubts/regrets for not doing them sooner'. P 67, Did not meet criteria for (C)PTSD
**Structural barriers to empathetic care** NHS and private clinics' resources (waiting areas, rooms, equipment, staffing, staff training) and logistical limitations (lack of continuity of care, shared wards, rooms, walkways and staff with ob-gyn and maternity wards) compound the feelings of poor care and lack of compassion towards patients who end unsuccessful treatment.	65 (16%)	'I saw a different staff member each time and they didn't know or understand my case and didn't sound invested'. P 136, Did not meet criteria for (C)PTSD<br>'Poor management of hospital set ups when miscarrying or having fertility treatment as in with maternity'. P 99, Did not meet criteria for (C)PTSD
**Poor communication and information provision at all stages of treatment** Limited communication and preparation before, during and after IUI and IVF cycles, a lack of transparency about treatment success rates, and poor information sharing with patients. Staff's lack of knowledge mentioned as well.	64 (15%)	'Not being given information on problems in health care system at the time. Bounced around waiting lists. GP recommend I stay with NHS and didn't know about waiting lists. Delays cost me in relation to age and ability to gather own eggs—time is important in fertility and I felt this was not appreciated by the professionals or to give me the information I needed to make alternative decisions and working that out cost me time and money'. P 122, Did not meet criteria for (C)PTSD<br>'Having minimal consultation at the clinic, feeling rushed through the process. I feel that our one shot of funded treatment was wasted due to our lack of understanding of the process and how much it was taken from us. We were deer in the headlights with no one really telling us any information that we could understand or process'. P 124, Met criteria for (C)PTSD
**Lack of psychosocial support** Clinics provide very limited guidance and support for the psychosocial impacts of infertility and its treatment (i.e. counselling, psychosocial care), resulting in patients' overwhelm and impaired ability to cope with treatments' setbacks and challenges.	42 (10%)	'I had a miscarriage after my first IVF and received no support. I had a difficult pregnancy after my second IVF with risk of miscarriage again and no support'. P 7, Met criteria for (C)PTSD<br>'(…) and just the lack of support making me feel more alone and isolated and hopeless'.<br>P 566, Did not meet criteria for (C)PTSD
**Lack of empathy and compassion** Experiences of being treated in an insensitive way and without compassion by healthcare professionals	32 (8%)	'I was told my pregnancy losses were "a normal part of IVF". I was told "we can't make new eggs and so there is little we can do to help'. P 258, Did not meet criteria for (C)PTSD<br>'Staff having no understanding of the trauma making flippant comments towards us'. P 584, Did not meet criteria for (C)PTSD

Continued

Supplementary Table S9. Continued

Themes and categories description	Total sample *k* (%) /415 codes	Illustrative quotes
		'I felt the nurse who confirmed our missed miscarriage was dismissive and not very empathetic. I felt like we were an inconvenience to her. She showed us the screen and didn't talk through what had happened or enquire about mine or my partners mental state. She also began typing on her computer and left me on the examination table naked until I had to prompt her whether I could get dressed, as she had forgotten about me'. P 199, Met criteria for (C)PTSD
**Care during Covid-19 Pandemic** Fertility care during the Covid-19 Pandemic was reduced, severely limited or stopped altogether. Couples usually were unable to attend the appointments together, resulting in increased anxiety, stress and discomfort.	25 (6%)	'COVID didn't help; I was unable to be there for large parts of my wife's treatment. Even since COVID, for our second miscarriage, they tried to kick me out of the hospital overnight. I begged them to let me stay, and I'm pleased I did as my wife lost a lot of blood that night'. P 248, Did not meet criteria for (C)PTSD<br>'Throughout COVID restrictions meant that most appointments were done solo or over the phone. I understand why this had to be the case, but not having my partner there was difficult'. P 45, Met criteria for (C)PTSD
**Microaggressions**<br>Experiences of indirect, subtle or unintentional discrimination that communicate healthcare professionals' biases towards fertility patients. Participants reported instances of BMI shaming, being ignored, agism and misogyny, among others.	23 (6%)	'Referral to BMI constantly, inhumanly'. P 90, Met criteria for (C)PTSD<br>'We were told that if we'd started trying for a baby earlier, we wouldn't be having infertility problems. That then makes you feel awful for wanting to focus on your career and get married before we started trying for a baby'. P 9, Met criteria for (C)PTSD<br>'Medical professionals only talking to me not my husband'. P 162, Did not meet criteria for (C)PTSD
**Language and manner of speaking**<br>Experiences of being spoken to in an insensitive way, encompassing both the choice of language and the manner of speaking, without showing sympathy for fertility patients' feelings, and refusing to give importance to their experiences.	16 (4 %)	'Bluntness of some consultants. 30 second video call to tell my partner and I that he was infertile. Other HCPs were great'. P 266, Did not meet criteria for (C)PTSD<br>'NHS Mental health practitioner that was apparently an infertility counsellor made some very insensitive comments about how stress couldn't impact fertility because 'women in war zones get pregnant all the time and women in the holocaust still got pregnant' such awful comments to make to someone that was experiencing high levels of stress due to infertility diagnosis. My stress and anxiety were dismissed'. P 133, Did not meet criteria for (C)PTSD
**Fertility care is a business**<br>Private sector fertility clinics are a part of a growing industry where not only profit is made at the cost of patients' experience, their wellbeing and health, but also care is dismissive	14 (3%)	'The first clinic we attended, we got the impression of a business more so than a care facility. Just felt we were another number passing through, with this test and that test offered and extras put on the table that you are afraid to say no to because you have so much depended on it'. P 55, Met criteria for (C)PTSD<br>'Charging me for blood tests to confirm my baby was gone—even though they were sending me to an NHS hospital'. P 263, Did not meet criteria for (C)PTSD<br>'The lack of contact from the clinic once I couldn't afford any more treatment'. P 83, Met criteria for (C)PTSD
**Theme**<br>**Lack of control**<br>Fertility care is characterized by an experience of severely limited control over several aspects of the treatment.<br>**Categories are:**	67 (16%)	
**Treatment is not flexible nor person-centred**<br>The absence of individualized fertility treatment in both NHS and private clinics. Lack of opinions on treatment leading to feelings of frustration and disappointment.	27 (7%)	'The lack of help they give in general, it's: pay this money, do this protocol oh it doesn't work ok you're the issue not us or the protocol'. P 46, Met criteria for (C)PTSD<br>'Inflexible rules'. P 12, Did not meet criteria for (C)PTSD<br>'GPs and Consultants not communicating sensitively or centring me as a person beyond just a medical case'. P 150, Did not meet criteria for (C)PTSD
**Waiting**<br>Having to wait at all levels of fertility treatment and experiencing delays in treatment, also due to the Covid-19 pandemics.	22 (5%)	'Again, it's the continuous delays for everything. It's such a long process the last thing you want to hear after a failed transfer is, yes, it will probably be another 3 months till your next transfer'. P 296, Did not meet criteria for (C)PTSD<br>'The length of wait in between seeing professionals is quite ridiculous. It makes everything so much worse because you spend months not knowing what to do or what will happen next'. P 44, Did not meet criteria for (C)PTSD
**Lack of access to treatment**<br>The high treatment cost and funding limitations mean that fertility care is only available to those who can afford it.	18 (4%)	'Being left feeling that the clinic had it within their power to do more but the financial implications mean we cannot continue treatment'. P 278, Met criteria for (C)PTSD<br>'When you invest that much money in trying to have a baby, it makes your inability to do so far worse to cope with because you have invested so much of your future'. P 4, Did not meet criteria for (C)PTSD
