# Supplementary material for: Experiences of infertility-related traumatic events and their association with symptoms of Post-Traumatic Stress Disorder (PTSD) and Complex PTSD: results from a mixed-methods online survey
Source: Hum Reprod. 2026 Mar 12;41(5):772–85. doi: 10.1093/humrep/deag030 (PMC13139654; doi:10.1093/humrep/deag030)
Supplement: deag030_Supplementary_Table_S10 [file deag030_supplementary_table_s10.pdf]

Human Reproduction, 2026, 1–1
https://doi.org/10.1093/humrep/deag030
Supplementary Data

Supplementary Table S10. Qualitative theme *Lack of control*, its categories (in white), number of codes (k), and proportion (%) of total codes.

**Theme**<br>**Lack of control**<br>Fertility care is characterized by an experience of severely limited control over several aspects of the treatment.<br>**Categories are:**	67 (16%)	
**Treatment is not flexible nor person-centred**<br>The absence of individualized fertility treatment in both NHS and private clinics. Lack of opinions on treatment leading to feelings of frustration and disappointment.	27 (7%)	'The lack of help they give in general, it's: pay this money, do this protocol oh it doesn't work ok you're the issue not us or the protocol'. P 46, Met criteria for (C)PTSD<br>'Inflexible rules'. P 12, Did not meet criteria for (C)PTSD<br>'GPs and Consultants not communicating sensitively or centring me as a person beyond just a medical case'. P 150, Did not meet criteria for (C)PTSD
**Waiting**<br>Having to wait at all levels of fertility treatment and experiencing delays in treatment, also due to the Covid-19 pandemics.	22 (5%)	'Again, it's the continuous delays for everything. It's such a long process the last thing you want to hear after a failed transfer is, yes, it will probably be another 3 months till your next transfer'. P 296, Did not meet criteria for (C)PTSD<br>'The length of wait in between seeing professionals is quite ridiculous. It makes everything so much worse because you spend months not knowing what to do or what will happen next'. P 44, Did not meet criteria for (C)PTSD
**Lack of access to treatment**<br>The high treatment cost and funding limitations mean that fertility care is only available to those who can afford it.	18 (4%)	'Being left feeling that the clinic had it within their power to do more but the financial implications mean we cannot continue treatment'. P 278, Met criteria for (C)PTSD<br>'When you invest that much money in trying to have a baby, it makes your inability to do so far worse to cope with because you have invested so much of your future'. P 4, Did not meet criteria for (C)PTSD
