# Supplementary material for: Experiences of infertility-related traumatic events and their association with symptoms of Post-Traumatic Stress Disorder (PTSD) and Complex PTSD: results from a mixed-methods online survey
Source: Hum Reprod. 2026 Mar 12;41(5):772–85. doi: 10.1093/humrep/deag030 (PMC13139654; doi:10.1093/humrep/deag030)
Supplement: deag030_Supplementary_Table_S11 [file deag030_supplementary_table_s11.pdf]

OXFORD
human reproduction

Human Reproduction, 2026, 1–2
https://doi.org/10.1093/humrep/deag030
Supplementary Data

Supplementary Table S11. Qualitative theme *Suggested additional support*, its categories, number of codes (*k*), and proportion (%) of total codes.

Theme and categories description	Total sample *k* (%) /625 codes	Illustrative quotes
**Theme: Suggested additional support** Patients made several suggestions for additional support, focusing on communication and preparation for treatment could be managed, provision of psychosocial support (counselling, signposting, resources), structural and organizational factors, and patient-centred care. **Categories are:**	625 (100%)	
**Transparent two-way communication** Communication and preparation before, during and after IUI and IVF cycles with transparency about treatment success rate and impact of IVF. Empower patients to talk about their feelings and feel heard.	140 (22%)	'Acknowledge it. My pain and experience were almost made to feel like I was the only person who'd experienced it being that painful. Almost as though I was being a drama queen'. P 274, Did not meet criteria for (C)PTS<br>'A clearer understanding of the impact IVF can have whether successful or not and to be told from the beginning the small chance of IVF even working. I think you start IVF with an unrealistic view that it will work and that is not always the case'. P 9, Met criteria for (C)PTSD<br>'Better transparency so I didn't get traumatised as much in the first place!'. P 45, Met criteria for (C)PTSD
**Counselling support** Infertility specific counselling to help manage the psychosocial impact of treatment. There is a need for a safe space to talk about their experiences that is offered at the point of need.	139 (22%)	'I believe the counselling services should automatically be for all patients and not be a device you have to call and refer yourself. That was a hard task in itself for me. It made me feel that I used it because I was failing'. P19, Met criteria for (C)PTSD<br>'I think it would be good to give an appointment with the councillor as soon as you are referred for fertility treatment'. P79, Did not meet criteria for (C)PTS<br>'On-going access to fertility counselling after treatments finish, the comfortable space to talk openly about infertility without it being dismissed or 'offered' unhelpful advice/solutions like adoption. It felt very unhelpful for healthcare professionals then talking about their own parenting or infertility issues which resulted in children. Mostly an understanding from health-care professionals the enormity of infertility and especially childlessness and how it impacts on every aspect of our lives. Often dismissed and minimised'. P189, Did not meet criteria for (C)PTS
**Improvement in organisational and logistical issues** Improvement in management and resources of fertility clinics to facilitate better communication, easier access to resources and doctors and reduce communication errors. Psychosocial resources should be easy to access with impartial gatekeepers. This aims to alleviate patients feeling of isolation, anxiety, miscommunication and challenges accessing support.	102 (16%)	'Better systems in the first place to prevent a lot of the distress that was caused through long wait times and admin errors'. P 29, Met criteria for (C)PTSD<br>'More access to fertility treatments. Private clinics are so busy you just feel very alone and like you're on a conveyor belt. Even if you go to your GP they are so removed from the private clinic and will just revert you back there for any advice. But there just isn't enough staff in clinics, it's impossible to get through on the phone and you could be waiting days for a call back. Also, it's all done via admin or nurses, very little access to your actual doctor'. P 50, Did not meet criteria for (C)PTS<br>'Clearly state that it won't impact my ability to carry on with IVF. I'm concerned that by acknowledging the state of my mental health due to infertility will make them think I'm unfit to be a parent and that it would impact my treatment. Some more information about in what way they can support would also be helpful'. P289, Met criteria for (C)PTSD<br>'Different waiting rooms in hospitals if you've previously had a loss. I honestly don't think that there is anything worse than having to gear yourself up for a scan (which is incredibly traumatic after a loss) and to then have to sit in a waiting room with every other pregnant person'. P 191, Met criteria for (C)PTSD

Continued

Supplementary Table S11. Continued

Theme and categories description	Total sample k (%) /625 codes	Illustrative quotes
**Client-centred tailored psychosocial protocol**<br>A client-centred protocol for healthcare professionals to standardize care and allow for regular check in points. This aims to ensure mental health is on par with psychical health and has its own protocol to follow in terms of a treatment plan.	73 (12%)	'Better aftercare would have improved our experience in general. Making sure that advice/protocol was standardised, so that we didn't receive one set of instructions from one person and then told to follow a different protocol by another'. P 229, Met criteria for (C)PTSD
**Psychosocial support resources.**<br>A variety of resources that can support people with different needs at different stages to feel heard, understood and not alone.	70 (11%)	'The best thing I've found in the way of support, has connecting with other people going through fertility issues. I did this off my own back by hosting walk and talks, advertised at my fertility clinic and online'. P 253, Met criteria for (C)PTSD<br>'When we had the miscarriage, we were given a piece of paper with the Tommy's number on it but other than that we've not been offered any support. When you have your consultations or are doing your tests with the GP it would have been helpful to be pointed in the direction of charities that can offer advice, 'influencers' to follow who talk about their journey and counsellors trained in infertility'. P 70, Met criteria for (C)PTSD
Trauma informed staff<br>*All staff that are involved in fertility, antenatal and postnatal care should be aware of the impact of infertility and its treatment, as well as its potential for traumatising patients.*	*65 (10%)*	'Understanding the signs and symptoms of trauma, and things that can be put in place to help. If early signs are noticed, then this could stop it escalating to more serious trauma with the right support?' P 102, Did not meet criteria for (C)PTS<br>'For hospital to staff to have a better understanding of how infertility and loss can cause extreme anxiety and health concerns'. P 191, Met criteria for (C)PTSD<br>'More understanding from midwives about how hard infertility and miscarriage are and recognition of the anxieties this can cause'. P 201 Met criteria for (C)PTSD<br>'Some understanding of why a pregnancy as a result of fertility treatment might be more anxiety provoking. I didn't need referral to mental health services, but there were no online or paper resources for sub-clinical anxiety that were specific to fertility treatment pregnancy'. P 42, Did not meet criteria for (C)PTSD
Not sure<br>*Unsure of what support could be helpful*	*19 (3%)*	'I don't know what they could possibly say to help'. P199, Met criteria for (C)PTSD
Barriers to support<br>*Struggling to access support, unclear referral pathways, cost of support and unable to access through NHS, type of support offered was limited/unhelpful*	*17 (3%)*	'Private clinics are so busy you just feel very alone and like you're on a conveyor belt. Even if you go to your GP they are so removed from the private clinic and will just revert you back there for any advice. But there just isn't enough staff in clinics'. P50, Did not meet criteria for (C)PTS<br>'They [fertility clinics] offer counselling but you only get 1 or 2 on the NHS the rest you have to pay for. £40 a session is a lot of money'. P537, Did not meet criteria for (C)PTS
Support to be physically ready for IVF<br>*Support plans to help stay physically fit and healthy to aid with the IVF process.*	*4 (1%)*	'Longer-term support (not just 3 sessions), help with nutrition, keeping fit (to prepare body for another round of IVF), possible free acupuncture as this has proven to have benefits'. P 164, Did not meet criteria for (C)PTSD

© The Author(s) 2026. Published by Oxford University Press on behalf of European Society of Human Reproduction and Embryology.
This is an Open Access article distributed under the terms of the Creative Commons Attribution License (https://creativecommons.org/licenses/by/4.0/), which permits unrestricted reuse, distribution, and reproduction in any medium, provided the original work is properly cited.
Human Reproduction, 2026, 00, 1–28
https://doi.org/10.1093/humrep/deag030
Original Article
